# Inverse Stochastic Resonance in Cerebellar Purkinje Cells

**DOI:** 10.1371/journal.pcbi.1005000

**Published:** 2016-08-19

**Authors:** Anatoly Buchin, Sarah Rieubland, Michael Häusser, Boris S. Gutkin, Arnd Roth

**Affiliations:** 1 Group for Neural Theory, Laboratoire des Neurosciences Cognitives, École Normale Supérieure, Paris, France; 2 Institute of Physics, Nanotechnology and Telecommunications, Peter the Great St. Petersburg Polytechnic University, Saint Petersburg, Russia; 3 Center for Cognition and Decision Making, Department of Psychology, NRU Higher School of Economics, Moscow, Russia; 4 Wolfson Institute for Biomedical Research and Department of Neuroscience, Physiology and Pharmacology, University College London, London, United Kingdom; Radboud Universiteit Nijmegen, NETHERLANDS

## Abstract

Purkinje neurons play an important role in cerebellar computation since their axons are the only projection from the cerebellar cortex to deeper cerebellar structures. They have complex internal dynamics, which allow them to fire spontaneously, display bistability, and also to be involved in network phenomena such as high frequency oscillations and travelling waves. Purkinje cells exhibit type II excitability, which can be revealed by a discontinuity in their f-I curves. We show that this excitability mechanism allows Purkinje cells to be efficiently inhibited by noise of a particular variance, a phenomenon known as inverse stochastic resonance (ISR). While ISR has been described in theoretical models of single neurons, here we provide the first experimental evidence for this effect. We find that an adaptive exponential integrate-and-fire model fitted to the basic Purkinje cell characteristics using a modified dynamic IV method displays ISR and bistability between the resting state and a repetitive activity limit cycle. ISR allows the Purkinje cell to operate in different functional regimes: the all-or-none toggle or the linear filter mode, depending on the variance of the synaptic input. We propose that synaptic noise allows Purkinje cells to quickly switch between these functional regimes. Using mutual information analysis, we demonstrate that ISR can lead to a locally optimal information transfer between the input and output spike train of the Purkinje cell. These results provide the first experimental evidence for ISR and suggest a functional role for ISR in cerebellar information processing.

## Author Summary

How neurons generate output spikes in response to various combinations of inputs is a central issue in contemporary neuroscience. Due to their large dendritic tree and complex intrinsic properties, cerebellar Purkinje cells are an important model system to study this input-output transformation. Here we examine how noise can change the parameters of this transformation. In experiments we found that spike generation in Purkinje cells can be efficiently inhibited by noise of a particular amplitude. This effect is called inverse stochastic resonance (ISR) and has previously been described only in theoretical models of neurons. We explain the mechanism underlying ISR using a simple model matching the properties of experimentally characterized Purkinje cells. We found that ISR is present in Purkinje cells when the mean input current is near threshold for spike generation. ISR can be explained by the co-existence of resting and spiking solutions of the simple model. Changes of the input noise variance change the lifetime of these resting and spiking states, suggesting a mechanism for a tunable filter with long time constants implemented by a Purkinje cell population in the cerebellum. Finally, ISR leads to locally optimal information transfer from the input to the output of a Purkinje cell.

## Introduction

Understanding the way neurons integrate synaptic inputs and provide appropriate outputs are crucial steps in the process of understanding neural circuits and relating their function to the function of specific brain areas. The cerebellar circuit is believed to be involved in ongoing motor control and motor learning, and an increasing amount of evidence suggests that it is the primary location of motor memories [1]. Purkinje neurons play a central role in the cerebellum, as they gather thousands of excitatory and inhibitory synaptic inputs from the molecular layer and provide the sole output of the cerebellar cortex. Describing and modeling the spiking response of Purkinje cells to synaptic inputs is therefore central to understanding cerebellar information processing.

Purkinje cells are spontaneously active even in the absence of synaptic input [2, 3]. It has been proposed that this notable intrinsic property is tightly linked to their type II excitability [4], [5] which is manifested by the non-zero minimum firing frequency in response to tonic current injection, and the characteristic discontinuity in the frequency-current relationship. This property is thought to be due to voltage-gated ion channels, such as resurgent sodium currents [6] or hyperpolarization-activated currents (*I*_*h*_) [4], which are active at rest. Such intrinsic mechanisms also underlie the ability of Purkinje cells to switch between spontaneous firing (up states) and quiet periods (down states). These have been observed *in vitro* [4, 7] and *in vivo* in anaesthetized [8] and awake animals [9]. The transition between these two states can be controlled by climbing fiber synaptic input [8, 9] or by molecular layer interneuron input [10]. However, the presence of these up and down states in awake animals has been controversial (see Discussion).

While the existence of up and down states is a consequence of the intrinsic biophysics underlying the type II excitability of Purkinje cells [5, 8] we can nevertheless expect synaptic input, specifically random noise-like synaptic inputs, to play an important role in patterning such firing behavior. An interesting dynamical phenomenon, inverse stochastic resonance (ISR), has been described recently in persistently periodically firing model neurons: variance-dependent inhibition of spiking in response to noise stimulation, including purely excitatory noise [11]. The unique defining characteristic of ISR is that inhibition of spiking shows a non-linear tuning with respect to input noise statistics, notably the variance. This phenomenon was first identified in computational models of bistable recurrent neural circuits [12], where the bistability was between persistent spiking and quiescent states and later shown in single bistable neuron models [11]. The key to ISR is the bistability between a steady state (“rest”) and a periodic activity state (“spiking”), a characteristic of systems with sub-critical Andronov-Hopf bifurcations. Interestingly, ISR appears to be robust to changes in noise color [13] and has also been demonstrated in spatially extended models of action potential propagation [14]. Further analysis showed that ISR should be a generic phenomenon in dynamical systems with steady-state/limit cycle multi-stability, yet so far no direct experimental measurement of ISR has been reported (but see [15] for hints of ISR in squid axons). Here we provide, to the best of our knowledge, the first experimental characterization of ISR in neuronal responses.

In this study we demonstrate ISR experimentally in Purkinje neurons and study its implications for cerebellar information processing. We find that Purkinje cells recorded in cerebellar slices show clear evidence for ISR. Their firing is reduced or can even be stopped in response to noisy current injection, with a non-linear dependence of the firing rate on noise variance which is characteristic for ISR. We further demonstrate that an empirically-based adaptive exponential integrate-and-fire model, quantitatively parameterized to fit Purkinje cell data using a modified dynamic IV method, reproduces both the bistability and ISR behavior of Purkinje cells. Finally, we show that the optimal noise variance for ISR also yields a local maximum in mutual information between the input and output spike train. Under these conditions, ISR leads to optimal inhibition of self-sustained spiking and thus provides the highest information transmission capacity for transient synaptic stimuli.

## Results

### Purkinje cells exhibit inverse stochastic resonance (ISR)

**Fig 1:**
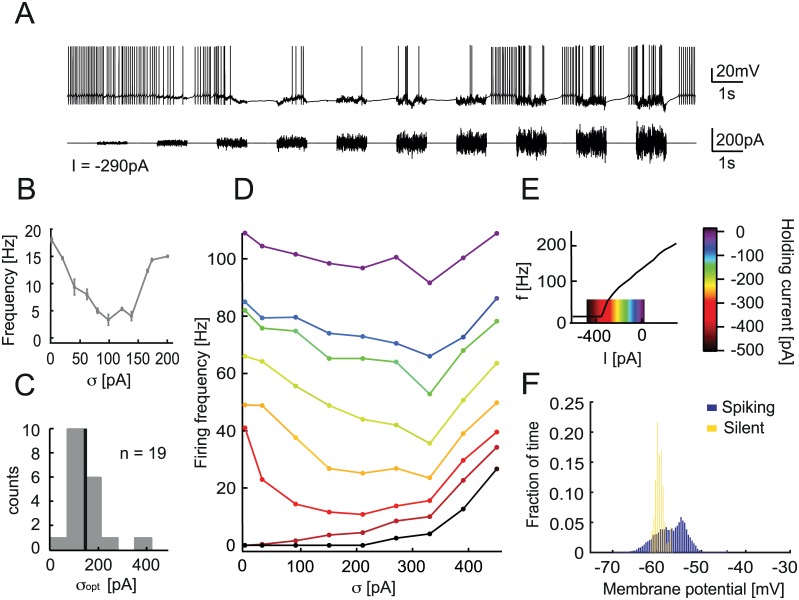
Cerebellar Purkinje cells show inverse stochastic resonance (ISR). A. Whole-cell patch-clamp recording from a Purkinje cell in a cerebellar slice, showing current injection of 1 s noise waveform periods with increasing amplitude, and recorded membrane potential *V*_*m*_. Holding current is *I* = −290 pA. The firing rate of the Purkinje cell (PC) is reduced for intermediary noise amplitude. B. Firing frequency during 1 s noise injection vs. noise amplitude *σ* corresponding to the trace in A. Error bars indicate standard deviation. The firing rate is minimal for *σ* = 100 pA. C. ISR is observed in all Purkinje cells tested. Summary of optimal noise amplitude σ = 152.60 ± 64.42 pA (n = 19). D. ISR curve of a different PC, generated with a current injection protocol of continuously changing noise amplitude and for a series of holding currents, exploring the full range of the *f-I* curve (E). The firing rate is most reduced when the cell is hyperpolarized to the edge of the *f-I* curve step. The optimal noise amplitude for inhibition of firing is σ = 200 pA. E. Frequency vs. current generated with 1 s step current injections. The color code corresponds to the region explored for the ISR curve in D. F. Membrane potential distributions computed from a somatic whole-cell patch-clamp recording from a Purkinje cell during injection of a stimulus current evoking transitions between spiking and silent states (A).

To test whether Purkinje cells (PCs) exhibit inverse stochastic resonance, we made patch-clamp recordings from PCs in rat cerebellar slices. We injected noisy current waveforms and observed the resulting firing behavior (Fig 1A). The noise protocol consisted of a series of ten 1 s noise periods alternating with 1 s rest periods. The noise waveform was generated by an Ornstein-Uhlenbeck process with time constant *τ* = 2 ms and increasing amplitude *σ* to represent the synaptic currents received by the cell (see Materials and Methods). We observed that the firing frequency of PCs is initially reduced in response to increasing noise amplitude. This counter-intuitive effect is characteristic of ISR, where the relationship between firing rate and noise amplitude has a minimum, or “tuning” (here at amplitude *σ* = 100 *pA*; Fig 1B). All Purkinje cells tested exhibited ISR, and the optimal noise level for firing rate inhibition for the population was *σ*_*opt*_ = 152 ± 64 pA (n = 19, Fig 1C). However, ISR is variable across cells, and some cells can be fully silenced in response to optimal noise amplitude injection (S1C Fig). In that case, the cell is generally silenced even during periods with no noise injection, if they follow periods with optimal amplitude noise. This shows that the firing rate in one interval is not only determined by the noise amplitude and mean (holding current), but also by the cell’s activity in the previous interval.

ISR appears to mediate transitions between a firing state and a silent state. To account for the resulting history dependence, we injected a noise waveform with continuously changing noise amplitude (Materials and Methods, S1E Fig), and generated ISR curves using only intervals in which the cell is initially in the firing state (Fig 1D, S1F Fig). We observed that the optimal noise amplitude *σ* consistently reduces the firing rate across a range of mean holding currents. However, the reduction is most pronounced when the cell is hyperpolarized relative to its resting membrane potential (holding current *I*_*in*_ = −290 pA for traces in Fig 1A and 1B). When the cell is hyperpolarized sufficiently to prevent firing, the noise injection then acts in the expected way and the firing rate only increases (Fig 1D, *I*_*in*_ = −400 pA). In this case, the noise amplitude needs to be large enough to bring the cell to threshold. We observe that there is an optimal holding current for which the cell’s firing rate is reduced the most (*I*_*in*_ = −350 pA, red), as there is an optimal noise level *σ*_*opt*_. This phenomenon is qualitatively similar to the previously reported ISR in the Hodgkin-Huxley neuron model. As Purkinje cells have type II excitability, we observe in particular that the optimal holding current corresponds to the region of the “discontinuity” in the f-I curve (red in Fig 1E). It appears therefore that the ISR phenomenon is linked to the bistability of Purkinje cells. Interestingly, Purkinje cells displayed a bimodal distribution of the membrane potential during transitions between up and down states induced by noisy current injections (Fig 1F).

### ISR parameters are related to Purkinje cell bistability

**Fig 2:**
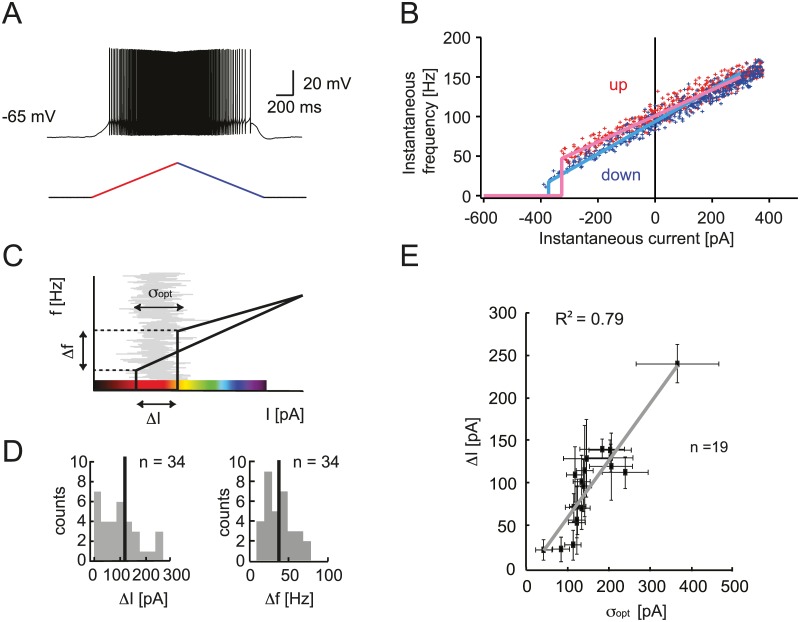
Experimental characterization of Purkinje cell bistability. A. Whole-cell patch-clamp recording from a Purkinje cell, showing a representative hysteresis measurement with slow current ramp injection (0.9 nA/s) ascending (red) and descending (blue), and the resulting PC membrane potential response. B. Instantaneous firing frequency and current for each spike. Linear fits of the ascending ramp (red) and the descending ramp (blue) are averages of 10 trials. C. Characterization of the hysteresis using the difference in frequency between first and last spike Δ*f* and difference in current Δ*I*. The color code illustrates the region explored for the ISR curve. Red corresponds to both the hysteresis and the optimal ISR region (Fig 1D and 1E). D. Distribution of hysteresis parameters across the population of recorded Purkinje cells. E. Correlation between the width of the hysteresis range Δ*I* and the optimal noise level for ISR *σ*_*opt*_. Error bars indicate standard deviation, *R*^2^ = 0.79 (n = 19).

We examined the link between the Purkinje cell intrinsic property of bistability and the modulation of firing by noise. Type II excitability is traditionally characterized by a step or discontinuity in the *f-I* curve (Fig 1E), as opposed to the continuous *f-I* curve of type I excitability [16, 17]. However, we observed that different Purkinje cells can show a wide range of type II behavior. The firing rate hysteresis in response to slow ramps of current allows a more precise characterization of this property than *f-I* curves [4]. Cells were held at −65 mV to prevent spontaneous firing and an ascending and descending (0.9 nA/s) ramp of current was injected (Fig 2A). The first spike occurs at a different instantaneous frequency and current than the last spike (Fig 2B). This hysteresis illustrates that the cell is in a different condition during the ascending and descending phases of the ramp.

We quantified the difference between the instantaneous frequency of the first spike (ascending) and the last spike (descending) (Δ*f*) and the difference of injected current for first and last spike (Δ*I*; Fig 2C). Across a population of Purkinje cells, we observed a wide range of hysteresis parameters Δ*f* = 38.17 ± 19.12 Hz, Δ*I* = 110.28 ± 84.57 pA (mean ± S.D., n = 34). Hysteresis as characterized by these parameters offers a quantification of the degree of bistability of individual Purkinje cells. Using the same color code for the hysteresis plot (Fig 2C) and the ISR curve (Fig 1D), we highlight the suspected link between the two phenomena. The inhibition of firing in response to noisy input is indeed more pronounced when the cell is hyperpolarized to the hysteresis region where both spiking and rest states can exist. To illustrate this relationship empirically, we compared the hysteresis parameters with the parameters of ISR. We found a correlation between the width of the hysteresis range Δ*I*; and the optimal noise amplitude (*R*^*2*^ = 0.79; n = 19 cells) (Fig 2E). This suggests that the noise amplitude required for ISR is on the order of the difference in the holding currents at which the cell makes transitions from the firing to the silent state and vice versa.

### An adaptive exponential integrate-and-fire (aEIF) model describes Purkinje cell firing

We employed a reduced model approach to understand the relationship between the intrinsic property of bistability and ISR. Our choice of a minimal spiking neuron model, which can describe the bistability of simple spike output of Purkinje cells, was motivated by several requirements. The model should have sufficiently rich dynamics to account for the bimodal behavior of Purkinje neurons, and be of sufficiently low dimensionality to allow analytical insights. The adaptive exponential integrate-and-fire model (aEIF) was chosen since it can reproduce a range of different firing regimes including type II excitability [16] and we can quantitatively fit the parameters of the model to electrophysiological data from individual PCs. The aEIF also allows a detailed examination of the links between hysteresis, bistability and ISR. To quantitatively fit the model to the experimental data, we used the dynamic IV method [18] for the parameters of the basic aEIF model together with a modified version of this method for the adaptation parameters (see Materials and Methods for details). Briefly, the dynamic IV method relies on accessing passive and active membrane mechanisms during physiological spike generation. It provides a simple representation of the ionic transmembrane current *I*_*m*_ as a function of the injected current *I*_*in*_ and membrane potential *V* by subtracting the capacitive current from *I*_*in*_.

$$I_{m}\left(V,\;t\right)=I_{in}\left(t\right)-C\frac{dV}{dt}+I_{noise}$$ (1)

**Fig 3:**
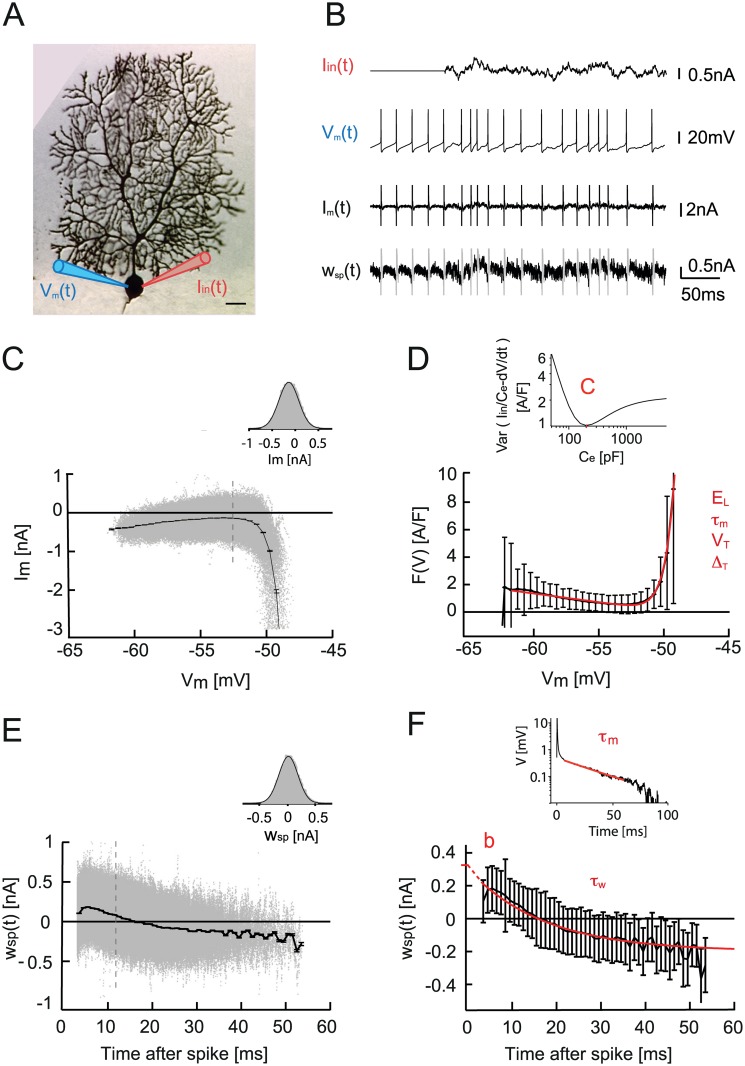
aEIF model fitting procedure to Purkinje cell experimental data. A. Double somatic whole-cell patch-clamp recording from a representative Purkinje cell: one electrode for current injection and one for voltage recording (scale bar, 100 *μ* m). B. Traces of injected noise current *I*_*in*_(*t*), recorded membrane potential *V*_*m*_(*t*), in a spontaneously active PC, calculated membrane current *I*_*m*_(*t*), and calculated spike-dependent adaptation current *w*_*sp*_(*t*). C. *I*_*m*_ vs. *V*_*m*_ and dynamic IV curve as the average over *V*_*m*_. Error bars indicate SEM. Inset, the distribution of data points at *V*_*m*_ = -52 mV is Gaussian. D. Fitting the dynamic IV curve F(*V*) = −*I*_*dyn*_/*C* with the EIF model function. Parameters are: resting potential *E*_*m*_, membrane time constant *τ*_*m*_, threshold potential *V*_*T*_, and spike slope factor Δ_*T*_. Error bars indicate SD. Inset, capacitance determination by minimizing the variance of *I*_*m*_. E. Spike triggered adaptation *w*_*sp*_(*t*) plotted versus time after the last spike. Error bars indicate SEM. Inset, the distribution of data points at *t* = 12 ms is Gaussian. F. The spike-triggered adaptation is fitted to a single exponential, with time constant *τ*_*w*_ and *w*_*sp*_ (t = 0) = b. Error bars indicate SD.

In order to mimic excitatory and inhibitory synaptic currents, the injected current was a sum of two Ornstein-Uhlenbeck processes with time constants *τ*_*fast*_ = 3ms, *τ*_*slow*_ = 10 ms [18]. Measuring the membrane potential using an electrode while simultaneously injecting current can be inaccurate because of the voltage drop across the electrode. Therefore, to measure the true membrane potential *V*, we performed double somatic patch-clamp recordings from PCs in slices (Fig 3A). The ionic current through the membrane *I*_*m*_ was calculated using Eq (5) and plotted against the voltage (Fig 3B and 3C). The distribution of the *I*_*m*_ data points is Gaussian for a given voltage (Fig 3C, inset), the dynamic IV curve is therefore defined as the average of *I*_*m*_ versus *V* (Fig 3D). The capacitance can be estimated by minimizing the variance of *I*_*m*_ within individual voltage bins (Fig 3D, inset) (see Materials and Methods). The dynamic IV curve can be readily transformed into an integrate-and-fire (IF) type neuronal model, where *F*(*V*) is a non-linear function of voltage.

$$F\left(V\right)=\frac{1}{\tau_{m}}(E_{L}-V+\Delta_{T}\text{exp}\left(\frac{V-V^{T}}{\Delta_{T}}\right)$$ (2)

The experimentally derived *F*(*V*) = −*I*_*dyn*_(*V*)/*C* curve can therefore be fitted with a function *F*(*V*) describing an exponential integrate-and-fire (EIF) model [19], with parameters membrane time constant *τ*_*m*_, resting potential *E*_*L*_, threshold potential *V*^*T*^, and spike slope factor *Δ*_*T*._ Average values for our PCs were: *C* = 195.4 ± 53.3 pF, *E*_*L*_ = −51.9 ± 1.9 mV, *V*^*T*^ = −54.1 ± 2.3 mV, Δ_*T*_ = 1.0 ± 0.2 mV, *τ*_*m*_ = 4.4 ± 1.2 ms (n = 7 cells).

It is interesting to note the difference between the dynamic IV of PCs and those previously reported for pyramidal cells [18]. The spontaneous, self-sustained, activity of PCs means that the IV dynamic curve sits above zero, and *V*_*m*_ never effectively reaches the resting potential *E*_*L*_. The aEIF model with these parameters is spontaneously active but it is not able to show type II excitability. To account for this essential property, we chose to extend the model with voltage-dependent adaptation. The method we used to fit the adaptation is inspired by the procedure used for fitting an aEIF model to synthetic data as described in [20]. Since the PC is spontaneously active, the classical dynamic IV method yielded a good approximation of the capacitance and reversal potential *E*_*L*_ (for more details see Supplementary information). However, the time constant determined by the dynamic IV method in Purkinje cells reflects the only fast time constant of a soma while the total membrane time constant, needed for the aEIF, depends on the large proximal dendrite as well. To compensate for this issue we estimate the full membrane time constant *τ*_*m*_ of Purkinje cell by fitting the voltage response to a short current pulse (0.5 ms, 1 nA), S2A Fig [21], see S1 Text. From this, we can estimate the value for the leak conductance *g*_*L*_ at the soma and proximal dendrite, using the membrane time constant and the capacitance *g*_*L*_ = *C*/*τ*_*m*_. We determine the subthreshold adaptation parameter *a* using the same approximation as in [20] (see Materials and Methods for more details), by subtracting the leak conductance *g*_*L*_ from the slope fitted on the linear part of the dynamic IV curve.

The voltage-dependent adaptation strength is the key parameter for type II excitability. To define it, we followed a method similar to the dynamic IV. To access the contribution of spike-triggered adaptation, we rearranged Eq (16) using the approximation (see Materials and Methods):

$$w_{spike}=-I_{in}-C\frac{dV}{dt}-\left(g_{L}+a\right)\left(V-E_{L}\right)$$ (3)

As *w*_*spike*_ is triggered at each spike, we plotted the estimated *w*_*spike*_ against the time since the last action potential (Fig 3E). The distribution of the data points is Gaussian for a given time (Fig 3E, inset). Therefore we defined the time course of the spike-triggered adaptation after a spike as the average of *w*_*spike*_ versus time. The curve can be fitted by a single exponential that yields an estimate of the time constant *τ*_*w*_. The adaptation parameters fitted to data from PCs were *a* = 36.1 ± 6.3 nS, *b* = 408.0 ± 128.0 pA, *τ*_w_ = 14.8 ± 6.3 ms (n = 7). The threshold parameters Δ_*T*_ and *V*^*T*^ are not influenced by adaptation, and were therefore used as fitted with the dynamic IV method.

### The aEIF model reproduces Purkinje cell bistability and ISR

**Fig 4:**
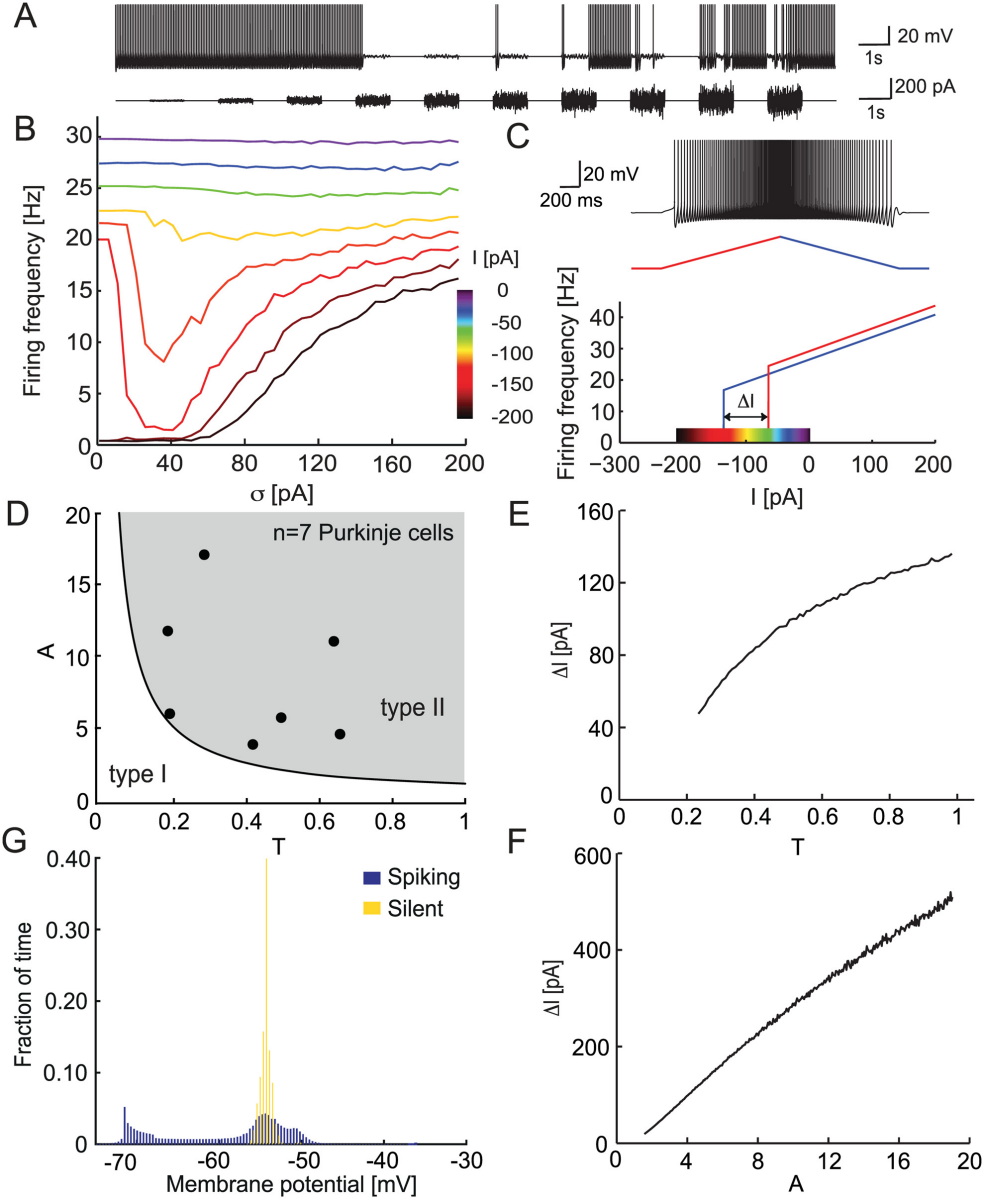
Hysteresis and ISR of the aEIF model. A Voltage response of the aEIF model to Ornstein-Uhlenbeck current noise injection with increasing amplitude. B. Mean firing rate of the aEIF model in response to current noise stimulation with amplitude *σ* and mean *I* (color code). C. Hysteresis of the aEIF model. Top, voltage response to ascending (red) and descending (blue) ramp of current. Bottom, instantaneous firing rate vs. instantaneous injected current. Color code is the same as in B. D. Parameter space of the rescaled aEIF model, white region: type I excitability, gray region: type II excitability. The 7 fitted cells are in the type II region. E, F. Dependence of the hysteresis size Δ*I* on the parameters *T τ*_*w*_/*τ*_*m*_ (E) and *A* = *a*/*g*_*L*_ (F). G. Membrane potential distribution in the aEIF model during spiking and silent states.

We used the empirically fitted adaptive exponential integrate-and-fire model (aEIF) to describe simple spike firing of a Purkinje cell in response to noise steps of increasing amplitude (see Fig 4A). The parameters of the model are quantitatively fitted for each cell using the modified dynamic I-V method as described above. All simulations were done for a parameter set representing a typical Purkinje cell.

**Fig 5:**
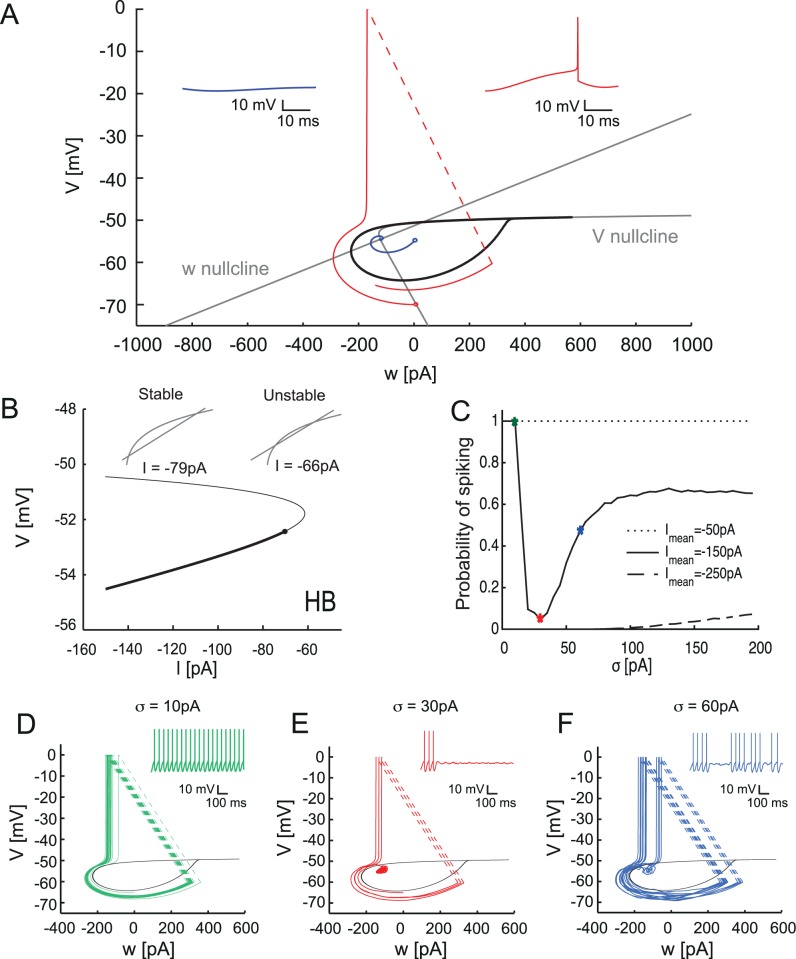
Bifurcation diagram and phase plane of the aEIF model. A. Phase-plane of the model. Gray lines are the null-clines of the model. Drop-like set (black) corresponds to the basin of attraction of a stable fixed point. Red and blue trajectories correspond to rest and spiking respectively (inset). B. Bifurcation diagram of the aEIF model. Solid and thin lines represent stable and unstable fixed points for different values of *I*. Inset shows the intersection of the null-clines before and after the Andronov-Hopf bifurcation (HB) point. Point corresponds to HB. C. Probability of spiking during the stimulation by noise with various means (compare Fig 4B). D, E, F. Phase plane of the model with the corresponding trajectories and voltage traces (inset) when stimulated by Ornstein-Uhlenbeck noise for 1000 ms with mean *I* = −150 pA and noise amplitudes *σ* = 10 pA, 30 pA and 60 pA.

The response of the model to current ramps showed clear hysteresis of the firing rate as a function of the current (Fig 4C). The mechanism for this hysteresis is fairly straightforward: the threshold for spike generation in the aEIF (and by extension in the PC) depends on the adaptation variable [18]. When the mean of the input current is set in the hysteresis region, the state of the cell becomes important for crossing the threshold. When the cell is in the spiking state, the intersection with the threshold takes place for more negative input due to the asymmetric shape of the attraction basin of the rest state (Fig 5A). This leads to longer interspike intervals when the system moves from spiking to rest during the downstroke of the ramp compared to the transition from rest to spiking during the upstroke.

Next we examined the firing rate responses of the model to current noise generated as in the experiment (Fig 4B). The mean of the input was chosen to be within the hysteresis range to reproduce the experimental conditions. When the mean input current was close to −150 pA the model demonstrated strong inhibition of firing by the injected noise. While extremely weak noise did not have much effect on spiking, noise with an optimal variance around 30 pA (that is still relatively small) efficiently switched the system from spiking to the rest state. Once an up-to-down transition occurred, the amount of current needed to elicit new spikes increased. This was due to the asymmetrical shape of the basin of attraction for the stable fixed point (see Section 5 below). Therefore, at the optimal noise variance the model preferentially remained in the resting state for an extended period of time. Interestingly, the variance of the noise optimal for spike-inhibition was unable to switch the model back from the rest state to the spiking state, hence we observe virtually no spontaneous down-to-up transitions. Noise with a sufficiently large variance was able to switch the system between spiking and rest. This variance-dependent inhibition by noise current led to dependence between the mean firing rate and noise variance that had a clear minimum (Fig 4B).

When the mean input current was outside the hysteresis region, this effect markedly decreased, or no ISR was observed. In the range above the hysteresis region (input current mean approx. −100 pA), this happened because the model was monostable, continuously spiking and the input noise resulted in a weak modulation of the firing pattern. When the input was below the hysteresis range, near −200 pA, the aEIF model preferentially stayed in the rest state at low noise values and spiked only if the input noise had a large enough variance (i.e. the spikes were directly evoked by the noise excursions above spike threshold).

To estimate the strength of ISR as a function of the model parameters and clarify the most important parameter combinations we rescaled the model (see Materials and Methods). This allowed us to significantly reduce the number of parameters. We estimated the bistability region of the rescaled aEIF model in terms of the hysteresis range *ΔI* = *I*_*up*_ + *I*_*down*_, i.e. the difference between threshold currents in the up-and-down ramp. The larger this difference, the stronger was the bistability and the more ISR would be present (meaning that the minimum of the firing rate vs. noise amplitude curve was deeper). According to our analysis the key parameters of the model are the voltage-dependent adaptation and the adaptation time constant. We found a sublinear dependence of the range of bistability on the adaptation time constant *T* (Fig 4E) and an approximately linear dependence on the adaptation parameter *A* (Fig 4F). This implied that the larger and the slower the adaptation, is the more prominent the difference between *I*_*up*_ and *I*_*down*_, and accordingly the larger the ISR range and the stronger the bistability. In conclusion, ISR is present for a wide parameter range as long as the model exhibits type II behavior.

Although the aEIF model can exhibit either type I or type II excitability depending on the parameter values (*A* and *T*) [16], our experimental results from 7 Purkinje cells showed that all measured neurons possess type II excitability in the model parameter space (Fig 4D). However, the size of the hysteresis region and the amplitude of the minimal firing frequency varied from cell to cell.

Note that while the aEIF model represents the bistability of simple spike output in Purkinje cells, it is a simplified model which does not capture bistability of the membrane potential [8] (Fig 4G), unlike a detailed biophysical model [22] (S5 Fig).

### Bifurcation analysis of the aEIF model

To understand the behavior of the model related to ISR, we examined the phase space of the model. The phase space of the model when the input mean is in the hysteresis (equivalently: bistability) regime consists of two areas: the basin of attraction of a stable fixed point and the spike generation area (Fig 5A). The drop-like set marked by the solid black line corresponds to the basin of attraction of a stable fixed point, the rest state. When a trajectory initiates in this region the system moves to the stable fixed point, e.g. the blue trajectory. Outside of this region all trajectories are spiking, for example the red trajectory. After each spike the system is immediately reset to *V*_*reset*_, so that the attraction basin is not crossed (dashed line). This implies that when the voltage of the model is transiently perturbed outside the droplet region, the model will continue spiking. This kind of behavior constitutes the bistability in the model, i.e. the coexistence of a spiking (limit-cycle) and a resting-state attractor for the same parameter set.

It is important to mention that the present model possesses discrete dynamics because voltage and adaptation variables are being artificially reset after each spike. In continuous models displaying ISR, such as the Hodgkin-Huxley model [23], the basin of attraction of the stable fixed point is also not crossed since after every spike the trajectory quickly moves to a close neighborhood of the resting state due to activation of the potassium current (delayed rectifier).

To obtain a more general picture of the mechanism underlying bistability we performed a bifurcation analysis of the model. Fig 5B shows the bifurcation diagram of the aEIF model (in the V-I plane) in the subthreshold regime (i.e. the bifurcation of the steady states). There are two fixed points corresponding to stable and unstable equilibria or fixed points in Fig 5B. When the input current *I* gradually increases, the rest fixed point loses stability via an Andronov-Hopf bifurcation (HB), which accounts for type II excitability. Due to the intersection of the nullclines there are two unstable equilibria after the HB point which merge and disappear at higher values of input current *I* (see insets). At higher input currents, there are no fixed points and there is only a spiking regime in the model. We can see that in the input ranges where ISR is present, the model has a stable equilibrium (a focus) and an unstable fixed point: this is a clear signature of the bistability as the upper unstable point corresponds to the voltage projection of the separatrix (see above) between the rest and the periodic processions toward the spiking threshold and the voltage reset.

To study the probability of transition to spiking we performed multiple numerical simulations when the model was stimulated by current noise with various means and amplitudes (variances). The resulting probability is shown in Fig 5C. The comparison of this spiking probability and the mean firing rate (Fig 4B) shows that these dependences have essentially the same shape. The similarity indicates that the mean firing rate represents the balance of the probabilities of down-to-up and up-to-down state transitions. When the mean current is within the bistability range, there is strong inhibition of spiking near 30 pA noise variance (red dot). For noise near 10 pA variance (green dot) the model preferentially stays in the spiking basin of attraction. However, when the noise amplitude becomes large (60 pA; blue dot) we observe random noise-driven crossings of the separatrix and transitions between spiking and rest. For still larger values of noise variance there is an increase in spiking probability, as it would be expected from a noise-driven threshold system. When the mean input current is beyond or below the bistability region *I* = [−100 pA, −200 pA], there is no significant inhibition of spiking (and so no ISR; Fig 5C, dashed and dotted lines). Fig 5D–5F show the trajectories in phase space for mean current within the bistability range and three different values of noise variance. In all cases initial conditions were initialized in the spiking region and then the model was stimulated with noise input with various amplitudes. This illustrates directly the phenomenon of ISR tuning as summarized in Fig 5C.

### Functional role of ISR

Bistability can significantly influence the output spike pattern and the response of a PC to external input. As discussed in the previous section, bistability and ISR can be explained using an aEIF model, implying that only the spike generating mechanism and a slow voltage-dependent adaptation are necessary for the phenomenon. In this section we investigate the role of bistability for processing of a transient external input when different levels of synaptic noise are present. Our goal is to examine if there is a link between ISR and the ability of the PC to respond to aspects of the incoming transient input (i.e. the signal).

**Fig 6:**
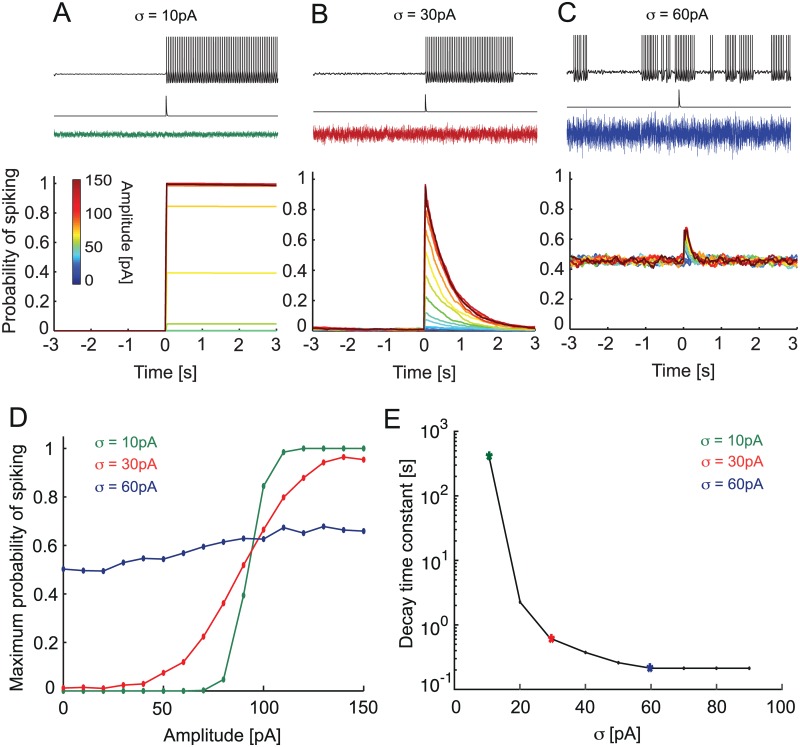
ISR transforms brief inputs into long-term firing states depending on background noise. A, B, C. Characteristic voltage traces of the aEIF model in response to a single synaptic excitatory input in the presence of different levels of background noise with amplitude *σ* = 10 pA, 30 pA and 60 pA. Bottom, corresponding probability of spiking for a range of input amplitudes (color code). D. Maximal probability of state transition vs. synaptic input amplitude for 3 background noise amplitudes, E. Decay time constant for the duration of the spiking state induced by a synaptic input of 100 pA. Remark: two data points corresponding to *σ* = 0 pA, 100 pA are not shown because for *σ* = 0 pA the duration of stimulus-induced spiking state is infinite, while for *σ* = 100 pA the duration of this state could not be distinguished from the firing baseline.

To highlight the role of ISR for the input-output relationship of PCs, the model was held in the bistability range by setting the mean input current to –150 pA, and it received two additional inputs: ongoing synaptic current noise and a brief excitatory current pulse (the signal) at a pre-set time (see Materials and Methods). To examine the input-output function of the model, we computed peristimulus time histograms (PSTHs, Fig 6). Since we were interested in understanding the role played by the ISR in the stimulus-induced transitions from the quiescent to the spiking state, initially the model was set in the rest state in all simulations.

In the case of low noise amplitude (Fig 6A), the model remained in the rest state before the arrival of the excitatory signal input, and hence the spiking probability was zero before the signal input; the basin of attraction of the rest fixed point is larger than the fluctuations induced by the noise. In this case the short synaptic excitatory input led to a clear sharp transition to the spiking state (Fig 6A). Once initiated, this spiking was not effectively inhibited by low-amplitude synaptic noise. Therefore the model remained tonically spiking after the application of the excitatory stimulus. In this situation the model acted as a latch, where the persistent spiking indicated that a stimulus has occurred at some point in the past. Had the model started in the spiking regime, the transient excitatory stimulus could switch it from spiking to rest (simulations not shown).

When the noise amplitude was optimal for inhibition (Fig 6B), the model remained mostly in the rest state before application of the excitatory stimulus due to the ISR. Therefore the probability of spiking before the arrival of the signal input was small. When the transient stimulus was applied it led to an increase of spiking probability and a finite number of periodic spikes were triggered. After the end of the transient stimulus, the spiking probability gradually decreased to zero due to noise-induced inhibition indicative of ISR. Hence at optimal ISR noise amplitude, the model produced a transient spiking response to the transient stimulus, yet with a duration of the response that was significantly longer than the stimulus itself. Moreover, the probability of the response was related to the stimulus amplitude. Hence, the timing of the input and its amplitude could be decoded from the PC spiking activity.

In the case of high noise amplitude the model randomly switched between spiking and rest (Fig 6C). This led to constant baseline probability of ~0.5 for spiking even in the absence of the stimulus. Once a stimulus was applied, it increased the probability of spiking compared to the baseline. The spiking probability decayed back to the baseline more rapidly than in the case of optimal noise amplitude for inhibition. Note that while there were spikes that were directly triggered by the transient stimulus, these spikes are hardly distinguishable from random spiking caused by the large amplitude noise. In this case the model acted neither as a latch nor is it able to signal the transient stimulus amplitude with any fidelity.

We summarize the noise-dependent effect on the response to the transient signal by plotting the peak probability of spiking as a function of the signal input amplitude, a signature of the input-output relation of the model (Fig 6D). At low noise amplitude the input-output relation is close to all-or-none, but with noise amplitude in the optimal ISR range, the response becomes more proportional to the input amplitude (a flatter sigmoid). Above the ISR range, the input-output relation is almost flat (little information in the output about the input). For the optimal noise amplitude (red curve Fig 6D) we see a sigmoid response behavior depending on the input amplitude. Below ~50 pA the model does not respond to the excitatory input by spiking because the input is not strong enough to bring the model out of the rest state. The peak probability saturates after ~125 pA amplitude. This means that beyond this value the model becomes insensitive to the amplitude of the input and will respond with the same amount of spiking even for larger amplitudes of the excitatory input.

Next we study the duration of a spiking state caused by signal input stimuli. Fig 6E shows the decay time constant of the spiking probability triggered by the transient input signal (Fig 6A–6C). For low values of synaptic noise, *σ* = 10 pA the model spikes for long periods of up to >1000 seconds. As noise amplitude increases, it significantly shortens the duration of the spiking induced by the stimulus. The reason for this effect is the following. As the noise variance approaches the ISR region (30 pA variance; green asterisk Fig 6E), the lifetime of the spiking state decreases, because the noise turns off the stimulus-evoked persistent firing after which the model stays quiet. When noise increases beyond ISR-optimal variance, the model starts generating spikes that are not evoked by the stimulus (Fig 6E, blue asterisk). This leads to lower values of the decay time constant as the model is switched ever more quickly between spiking and silent states by the noise.

We suggest that this may be a viable mechanism by which synaptic noise may control the duration of spiking responses induced by the external stimulus. In the low noise regime, spiking induced by the external stimulus would lead to long-lived spiking states, while in the presence of progressively stronger synaptic noise, the duration of the spiking state would become progressively shorter. In general, the probability of spiking in a PC population receiving the same single stimulus decays exponentially, but with different time constants set by the noise amplitude. Thus, in the optimal ISR noise regime the cell acts as a quasi-linear filter of the input, while in the low noise regime it acts as a memory device. Changing noise variance rapidly switches the cell from one mode to the other. Thus, the amplitude of the synaptic noise provided by ongoing parallel fiber input could provide a rapid mechanism to set the duration of a spiking state caused by external signal stimuli, such as a synchronous volley of parallel fiber input.

### ISR optimizes information transfer

**Fig 7:**
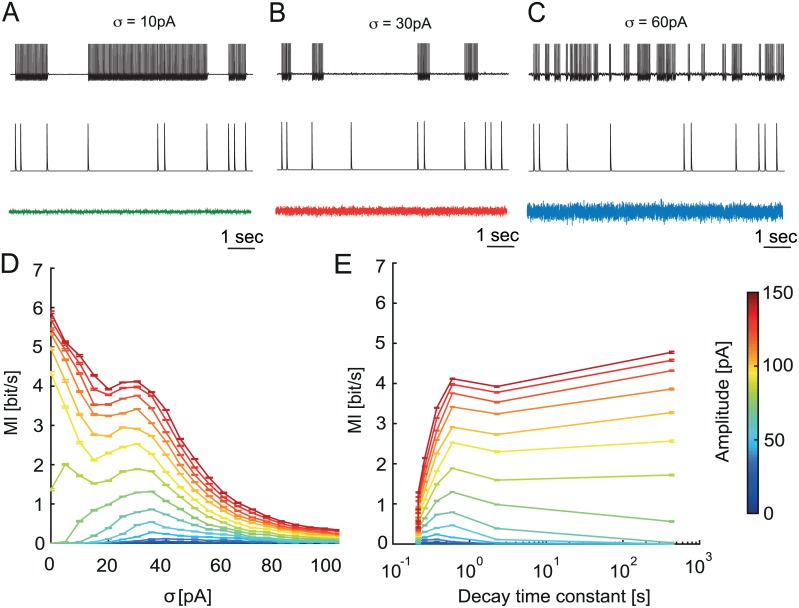
ISR leads to local optimum of mutual information between the input and output spike train. A, B, C. Voltage traces (top) of the aEIF model in response to series of excitatory synaptic inputs (middle, amplitude Am = 100 pA), in addition to background stimulation by noise with amplitude *σ* = 10 pA, 30 pA and 60 pA (bottom). D. Mutual Information (MI) of the input and output spike train of the aEIF model. E. MI as a function of the decay time constant (duration of a spiking state). Remark: two data points corresponding to *σ* = 0 pA, 100 pA are not shown (as in Fig 6E). For *σ* = 0 pA the duration of the stimulus-induced spiking state is infinite, while for *σ* = 100 pA the duration of this state could not be distinguished from the firing baseline.

We showed in the previous sections that ISR significantly modulates the persistent and stimulus-evoked firing in the experiment and the quantitative model of a PC. Here we examine how ISR affects the transfer of information across a PC for a series of input signals similar to those shown in Fig 6. To do so we take advantage of our quantitatively based aEIF model. The input for these numerical experiments consisted of synaptic noise with a parametrically adjusted amplitude and a Poisson signal spike train of 1 Hz mean rate. The noise input could represent spontaneous activity in the parallel fiber population, filtered by the dendritic tree of the Purkinje cell, while the signal input could represent sensory-driven clusters of synchronously active parallel fibers [24]. Depending on the synaptic noise amplitude, the state of the PC model can switch between periodic spiking (up state) and rest (down state) in response to the incoming inputs (Fig 7A–7C).

In the case of low variance noise, with the cell starting in the down state, the synchronous synaptic input can trigger an up state, which persists until the next one (Fig 7A). Given that the cell is in the up state, this next input then provokes the transition to the down state. This up-down transition will depend on the proper timing (or the relative phase of the spiking trajectory of the model) of an input: most efficient were inputs that were timed at a phase when the trajectory is close to the basin of attraction of a stable fixed point (Fig 5A). The down-up transitions did not depend on the specific timing of the input because the trajectory stayed in the resting state basin of attraction and did not have any definable phase in its noise driven fluctuations. Thus, a series of signal inputs caused continuous switching of the system back and forth between up and down states (Fig 7A).

When the noise variance was optimal for ISR (Fig 7B), the cell demonstrated a characteristic type of behavior. Due to the noise-induced inhibition of sustained firing, the model was in the resting state most of the time. Even when the initial conditions were chosen in the up state, the model quickly switched to the down state (Fig 6B). Once the external input was present, it brought the cell to spiking and the model stayed in the up state for ~1 sec (decay time constant, Fig 6E) followed by a transition to the down state because of the ISR effect. Thus, ISR ensured a resting baseline with minimal spurious spiking before the subsequent external inputs, making the firing output sparse and causing the onset of spiking to be related to the onset of the signal stimuli.

In case of strong synaptic noise, the cell is switching between up and down states even in the absence of external stimuli (Fig 7C). In this case an external stimulus leads to an increased probability of spiking compared to the baseline (Fig 6C). However, these additional spikes are rare compared to random firing caused by noise (Fig 7C). To study the efficiency of information transfer of the PC model we measured the mutual information rate (MI) between the input and output spike train for different levels of synaptic noise variance and input amplitudes. Fig 7D shows MI for different input amplitudes ranging from subthreshold (0–90 pA) to suprathreshold (100–150 pA) as a function of noise amplitude. A peak in MI appears around *σ* = 30 pA for a range of input amplitudes. This corresponds to the ISR noise variance optimal for inhibition. For the subthreshold input amplitudes this peak is global, while for the suprathreshold amplitudes it becomes local. It is interesting to note that MI has a local peak at ISR also for higher signal input rates (5 Hz; S4 Fig).

The presence of the MI peak for subthreshold input is the consequence of the classical stochastic resonance (SR) effect that has been extensively studied [25]. The contribution of ISR to this effect is the following. When the noise amplitude is optimal for inhibition, it provides a stable down state for a cell in the absence of an input. In this case the incoming input is amplified by noise due to the SR effect and will trigger spiking, i.e. produce an up state. Then due to the influence of ISR this stimulus-induced spiking will terminate before the next input. This leads to a strong temporal association between the input and output spike trains. Each input spike will correspond to a ~1 second (decay time constant, Fig 6E) spike train in the output with a high probability. After each input spike the cell will return to the down state due to ISR. This leads to the MI peak near the noise amplitude optimal for the ISR.

In the case of suprathreshold input, the MI was maximal for zero noise variance because a strong input could reliably trigger up and down transitions. In this case the noise plays mostly a disruptive role because it adds additional spurious output spikes unassociated with the input stimuli. However, in the presence of ISR the number of these spikes is smaller since synaptic noise prepares the baseline, setting the spontaneous spiking activity close to zero, thereby making the input and output spike train temporally associated. Therefore we observe a local peak of MI associated with ISR even for the suprathreshold input amplitudes.

To study the relationship between the duration of a spiking state and MI we combine the time constant estimation (Fig 6E) with our MI measure (Fig 7D). As shown in Fig 7E, a peak near 1 second spiking duration is present for all input amplitudes, which is the consequence of ISR. Similar to Fig 7D, this MI peak is global for the subthreshold input and becomes local for suprathreshold input. For subthreshold input, this means that the synaptic noise amplitude optimal for ISR corresponds to the optimal duration of the spiking state for information transfer measured by the output spikes, and for both types of input, long durations of the spiking state can coexist with high rates of information transfer.

## Discussion

We have characterized the effects of noise on the dynamical response regimes of cerebellar Purkinje neurons. We showed experimentally that simple spike firing in Purkinje cells can be efficiently inhibited by noisy input current if its variance is within a specific range, a phenomenon called inverse stochastic resonance (ISR). We then used an adaptive exponential integrate-and-fire (aEIF) model to quantitatively fit experimental data on subthreshold and spiking behavior of individual Purkinje cells using a modified dynamic IV method. For each cell, the resulting aEIF models exhibited parameter combinations generating bistable behavior. We found a good qualitative match between ISR measured experimentally in our Purkinje cells and in the aEIF model in terms of the hysteresis of the relation between firing rate and current, and the shape of the ISR curve. Analysis of the model revealed that ISR can be explained by the coexistence of a spiking and a resting state attractor. Using numerical simulations we showed that synaptic noise allows the Purkinje cell to switch between spiking up and silent down states, with their durations determined by the variance of the synaptic noise input. Our simulations further showed that ISR allows the PC to respond to transient inputs like a tunable filter, whose time constant can be set by the noise variance in a wide range, from a memory-toggle mode to a rapid filter mode. Finally, we show that a noise variance that is optimal for ISR leads to a local maximum of mutual information rate between the input and output spike train. These findings show that ISR is present in Purkinje cells and suggest possible roles for ISR in information processing in the cerebellar cortex.

### Purkinje cells display ISR

Traditionally, noise has been seen as enhancing neural responses by increasing the probability of crossing the spiking threshold, and increasing the reliability of the spike train [26]. Furthermore, a non-linear relationship was found between various measures of signal transmission (usually for a subthreshold stimulus) and the noise amplitude, a phenomenon known as stochastic resonance (SR). Much work on SR has identified the various stimulus and neuronal conditions for its existence, and potential functional roles (for a review see [25]).

A related, yet distinct phenomenon, where noise selectively decreases the probability of spiking, or converts persistent spiking activity into a short-lived transient followed by long-term quiescence has only recently been identified. This effect has become known as inverse stochastic resonance [27]. Initially described in bistable networks of spiking neurons [12], this phenomenon has subsequently been observed and analyzed in single neuron models, including in spatially extended ones [14]. At optimal noise amplitude, the duration of the transient intrinsic activity is minimized as the noise effectively quenches the neuronal response. Modeling work suggested that bistability is a necessary condition, while simulations of compartmental models proposed that ISR results from noise injection at the site of spike generation. Further work showed that colored noise is more efficient at producing ISR when compared to white noise [13], thereby hinting that synaptic noise may be particularly efficient at producing ISR in bistable neuronal systems. To our knowledge, the functional significance of ISR has not yet been analyzed, with the exception of [28] where ISR was suggested to play a role in limiting the duration of pathological working memories. Experimentally, signatures of noise-induced quenching of periodic activity were observed in the classical squid axon preparation [15], where noise injection effectively stopped repetitive spiking, yet no tuning properties of the noise amplitude were noted.

In this work, to our knowledge, we observed for the first time an experimental example of ISR in a neuronal preparation. One might ask the question of how widely are we expected to see this phenomenon? As we alluded above, the dynamical requirement for ISR, aside for varying noise levels, is the co-existence of a stable quiescent state and a repetitive firing limit cycle. One prominent dynamical path to such bistability is the subcritical Hopf bifurcation. This bifurcation is the basis for the so-called type II excitability, with the Hodgkin-Huxley model of the squid giant axon being a classical example. Hence, we might claim that any neuron that shows type II excitability should potentially exhibit ISR. In fact, Paydarfar et al. [15] recorded responses of a squid giant axon to noise injections and found that additive noise could quench repetitive firing. This is precisely a central signature of ISR. However, they did not look for noise to firing tuning and hence did not show ISR explicitly. What are the other examples of neurons that are likely to show ISR? Two cell types come to mind: the fast spiking interneurons and the cochlear hair cells. In an *in vitro* study of cortical fast spiking neurons, Robinson and colleagues [29] found clear signatures of type II behavior: a discontinuity in the f-I curve, a periodic voltage wobble around the resting potential (indicative of a stable focus) and an intermittent firing of periodic spikes (interleaved with periodic voltage oscillations) at somewhat depolarized potentials. All of these suggest a subcritical Hopf-driven excitability and hence we are likely to observe ISR in these cells. Cochlear hair cells have been argued to be a clear example of a cell that is governed by a Hopf bifurcation [30, 31]. While some of these cells appear to show signatures of a supercritical Hopf, a significant proportion of them have behavior compatible with the subcritical Hopf (hence should show hysteresis and bistability) [30, 32, 33]. We would thus venture to speculate that a subpopulation of cochlear hair cells should show ISR, and perhaps that a properly tuned noise maybe be potentially remedial for auto-emitted inner ear noise. Furthermore, Stiefel et al. [34] showed that with a sufficient level of the slow voltage dependent muscarine-sensitive potassium M-current, cortical pyramids show type II behavior and that the neuromodulator acetylcholine may toggle these cells from type II to type I. Hence it would be intriguing to test the subset of cortical pyramids with strong M-current for ISR. In summary, ISR should be observable in any cell type that shows bistable resonator behavior and should not be observed in cells that act as integrators (where we might expect standard SR).

In this study we present several lines of experimental evidence for ISR in cerebellar Purkinje cells. We found that simple spike firing in these neurons can be efficiently inhibited by current noise injections to the soma when the mean of the input current is in the subthreshold range (Fig 1D and 1E). To quantify this effect we measured the average firing frequency as a function of the input noise variance. We found the characteristic minima of the firing rate for particular noise amplitudes, which are optimal for ISR in different neurons (Fig 1B and 1C). Notably we observed that all Purkinje cells we studied displayed ISR (Fig 1C).

To identify the range of the mean input current for which ISR can be observed, we applied a symmetric current ramp protocol (Fig 2A and 2B). We found that the area of hysteresis between the ascending and descending instantaneous f-I curves indicates the range of mean currents at which ISR is present (Fig 1D). This hysteresis, which can be defined as the difference in currents (*ΔI*) between the first and last spike in response to the symmetric ramp, measures the degree of bistability of a particular cell. We found a strongly positive correlation between the degree of bistability of an individual Purkinje cell and the noise amplitude that is optimal for ISR in this cell. This argues for a strong link between ISR and the bistable behavior of Purkinje cells.

This hysteresis is also the reason why the occupancy of up and down states during noisy current injection is history-dependent (Fig 1A). Thus, simple noise injection protocols such as the one in Fig 1A cannot cleanly separate the steady-state ISR effect and the memory effect. We therefore switched to a noise injection protocol in which noise variance continuously changes (S1E Fig). The results obtained by brief constant-variance noise injections (Fig 1A) and the continuous noise protocol show a similar dependence of ISR on noise variance (S1B, S1D and S1F Fig).

The visibility of bistability in firing patterns of Purkinje cells recorded *in vivo* varies depending on experimental conditions, animal species and different regions of the cerebellum [35, 36]. There is evidence for up and down states in ketamine-anesthetized rats [37] and in awake behaving cats [9]. The presence of patterns and pauses in Purkinje cell simple spike activity *in vivo* could be interpreted to result in part from their bistable behavior [38]. On the other hand, recordings from Purkinje cells in the lateral and intermediate regions of the cerebellum concerned with arm movements do not show obvious up and down states [39, 40].

One possible solution of this controversy is that different degrees of visibility of Purkinje cell bistability can be explained by different neuromodulatory states in the cerebellum [37]. For example, serotonin can transform a tonically spiking Purkinje cell into one that displays bistability [4] whereas corticotropin-releasing factor can trigger down-to-up state transitions [41]. There is evidence that other biophysical mechanisms could regulate Purkinje cell bistability: for example, Bergmann glia could change the extracellular K^+^ concentration by Ca^2+^-dependent K^+^ uptake [42], thus modulating Purkinje cell excitability. Another possible explanation is that the properties of Purkinje cells could be different in various cerebellar zones [36, 43]. Also, our data demonstrates that Purkinje cells can exhibit various amounts of bistability (Figs 2C and 4D), which may represent diversity both within and across different zones of the cerebellum.

As we show in this study, changing the mean and the standard deviation of the synaptic input current is a very fast way to move Purkinje cells in and out of the range of bistability (Fig 1D). Thus, even if bistability is not always engaged and overtly visible *in vivo* [35], it is nevertheless likely that the underlying mechanisms are continuously present, and can influence Purkinje cell firing and network function in the cerebellum ([35], reply).

### ISR in the Purkinje cell model

Several detailed Purkinje cell models capturing the membrane properties as well as the anatomical structure of these neurons have been published, e.g. [21, 22]. Detailed models can exhibit a high degree of realism: for example, the model of De Schutter and Bower [22] correctly predicts ISR in Purkinje cells (S3 Fig), including the membrane potential distributions in the spiking and silent states (S5 Fig), while simple models could not capture this property (Fig 4G). Usually detailed models have numerous state variables describing the membrane potential and voltage-gated conductances in multiple compartments. The advantage of their biological realism is balanced by the large number of variables and parameters they contain. This high dimensionality does not allow a straightforward application of dynamical system theory to gain insight into the mechanisms of excitability, which makes the analysis of these models at times difficult.

In this research we therefore chose a reduced minimal model, the adaptive integrate and fire (aEIF) model, to study ISR and bistability. The advantage of this model is that it is well studied in terms of dynamical system analysis [16] and further relates to the normal form reductions of higher-dimensional models (including those with multiple compartments), making it in a sense a canonical model of spike generation. This allows us to use the dynamical system approach to analyze the model behavior. Despite the relative simplicity of the aEIF model, the estimation of the model parameters from experimental data is still a challenging task. Recently, the dynamic IV method [18] has been described to identify the parameters of aEIF models from intracellular recordings. Although it has not been used for spontaneously firing neurons, such as Purkinje cells, we show that it can be successfully applied to these neurons after necessary modifications of the dynamic IV method (see Materials and Methods).

We found that an aEIF model with the parameters provided by a modified dynamic IV method allows us to qualitatively reproduce ISR and the hysteresis of the firing rate (but not the bistability of the membrane potential [8]; S5 Fig) of the Purkinje cell. Remaining quantitative differences are likely to be due to the dynamic IV procedure, which does not allow us to estimate parameters related to the dendrite. This might be negligible for neurons with a relatively small dendritic tree [18], but the large dendritic tree of Purkinje cells strongly influences their responses even to somatic inputs [21, 44]. To compensate for this issue we have included a passive dendrite in the aEIF model and estimated the dendrite parameters (S2 Fig ISR and dendrite filtering, see S1 Text). We have found that the ISR effect is still present in the model with a passive dendrite, but the shape of the ISR curve becomes wider, which makes the model more consistent with the experimental data. We argue that using a two-compartment aEIF model would allow a quantitative match of the experimental ISR curve, but in a two-compartment aEIF model it is more difficult to precisely estimate the necessary parameters from somatic intracellular recordings.

The aEIF model has a very rich repertoire of dynamical states and can be tuned to reproduce the spiking behavior of many different neurons [18]. The relevant property for bistability and ISR is type II excitability, due to the presence of an Andronov-Hopf bifurcation. This bifurcation is responsible for the transition from the rest state to the spiking state and backwards. Crucially, it allows the model to have a spiking and a resting state attractor for the same parameter set. We show that the bimodal behavior of Purkinje cells as well as ISR can be explained by this bistability of the aEIF model solutions. When the initial conditions are set inside the rest state attractor, the model will come to the resting state, while in all other cases the model will continuously generate spikes (Fig 5A). The key parameter for bistability in the model is the mean of the input current. If it is in the bistable region of the f-I curve, then the model displays bistability most clearly in the presence of noise (Fig 4C).

When the model is in this regime, synaptic noise of particular amplitude is able to move the system preferentially to the basin of attraction of the rest state. This happens because the shape of the basin of attraction provides non-symmetric probabilities for up-to-down and down-to-up transitions. In the case of ISR, the probability of down-to-up transitions becomes very low, reducing the occupancy of the spiking state. This leads to a “latch” effect—once the system moves to the basin of attraction of the rest state, it cannot go back because the noise amplitude is not strong enough. Essentially this mechanism constitutes the main explanation for the ISR effect. Thus, ISR is possible only if the model is bistable. Rescaling of the model revealed the key parameter combinations responsible for bistability and ISR, such as adaptation and its timescale (Fig 4D, 4E and 4F).

### Functional consequences of ISR

The function of cerebellar Purkinje cells is often considered in the context of adaptive filter models of the cerebellum [45, 46]. A key property in this framework is the linearity of the input-output relation of Purkinje cells. Our data shows that above the minimum firing frequency, the relationship between the input current and the output firing rate is highly linear (Fig 2B). This is in line with previous findings [47], as well as with the linear phase response curve behavior of Purkinje cells [48, 49]. On the other hand, the step-linear shape of the f-I curve, which is a hallmark of the type II excitability of Purkinje cells, and the binary nature of the bistable behavior underlying ISR appear to contradict the idea that Purkinje cells perform linear transformations of their inputs. Since we show that Purkinje cells can operate in both regimes depending on the input current (Fig 2B), and the bistable behavior is stochastic, we suggest that this apparent contradiction can be resolved at the level of populations of Purkinje cells. We show that the size of the region of bistability varies in different Purkinje cells, as does the absolute position of the steps in the f-I curve. At the same time, the functional consequences of ISR on spike output will be felt over a wide range of values of the mean and variance of the input current in a given Purkinje cell (Fig 1D). It is also likely that different Purkinje cells in a population receive background synaptic input with different mean and variance, for example due to variability in the local structure of the feedforward inhibition circuit represented by molecular layer interneurons. This diversity in the intrinsic properties and the synaptic input in a population of Purkinje cells could result in an approximately linear input-output relation at the population level.

In addition to this approximate linearity, behaviorally relevant adaptive filters need to implement time constants that are much longer than the typical membrane time constants of Purkinje cells or other cerebellar neurons. The up and down states with their potentially long lifetimes could provide the necessary mechanism for filters with long time constants. Furthermore, our model suggests that the lifetime (in a single Purkinje cell) or the filter time constant (in a population of Purkinje cells) can be regulated by changing the variance of the input noise (Fig 6E). An alternative interpretation of the potentially long lifetimes of up and down states is that they could implement a form of short-term memory. This is in line with a recent study [50] which showed that bistability of Purkinje cells can increase their pattern storage capacity. Tunable Purkinje cell bistability could also be involved in generating the conditioned responses observed in [51].

We propose the following mechanism of up and down state transitions in ISR. When synaptic noise is optimal for inhibition (Fig 6B), the Purkinje cell preferentially stays in the resting state in the absence of specific signal stimuli. Once the neuron receives a strong external signal input, it brings the cell to the spiking state. The duration of this induced spiking can last up to several seconds, which is much longer than the membrane time constant of the Purkinje cell (Fig 6B). Eventually, the cell stops firing due to ISR and thus prepares for the next signal input. Thereby, when the noise variance is optimal for ISR, the Purkinje cell acts as a filter with a long time constant because a brief external input can trigger a long-lasting up state. In this case, input noise optimal for ISR plays two roles. First, it prepares the baseline for the next input due to the inhibition of spiking. Second, it sets the mean lifetime of the up state, and therefore the time constant of the filter.

For noise amplitudes below the ISR peak, the lifetimes of the up and down states increase further, leading to a different mode of operation of the Purkinje cell. In this mode, most transitions from up to down and from down to up are triggered by external signal inputs (Fig 6A). In this regime, the Purkinje cell acts like a toggle switch [8]. Thus, the amount of synaptic noise provided by parallel fiber input can tune Purkinje cell responses in a wide range between a toggle and a linear filter mode. This mode switch could occur at a very fast time scale, since the noise level in the parallel fiber population can change quickly. However, once the synaptic noise variance becomes too large (Figs 6C and 7C), most transitions between spiking and rest are triggered by the noise, and the Purkinje cell cannot reliably perform either of the two modes of operation.

To quantify how different input noise levels affect the information transmission capacity of the Purkinje cell, and to examine the potential role of ISR in information processing in the cerebellum, we estimated the mutual information between a signal input spike train and the output spike train of the Purkinje cell model at different levels of noise variance. We found that for the noise amplitude optimal for ISR, the mutual information rate has a local optimum, indicating that synaptic noise of particular amplitude can significantly enhance the transmission of information across the Purkinje cell to downstream neurons. In summary, ISR could provide a mechanism for setting both the time constants of temporal filters implemented by the firing of a Purkinje cell population, and the maximum rate of information that Purkinje cells can pass on to downstream targets.

## Materials and Methods

### Ethics statement

This study was performed in strict accordance with UK Home Office regulations. Experiments were carried out under Project License 70/7833 issued by the Home Office, which was issued following local ethical review (UCL AWERB), and under the relevant Personal Licenses issued by the Home Office.

### Slice preparation and patch-clamp recording

250–300 *μ* m thick parasagittal slices of cerebellar cortex were made from 18–24 day old Sprague Dawley rats by standard techniques [52]. Briefly, rats were anaesthetized with isoflurane for several minutes and decapitated in accordance with Home Office regulations. Slices were cut using a vibrating slicer (Leica VT1200S), after z-axis vibration was minimized to < 0.1 *μ* m. The slices were incubated in carbogen-saturated ACSF at 34°C for 30 min and then at room temperature for at least 30 min before use within four hours. Standard ACSF contained (in mM) 125 NaCl, 2.5 KCl, 2 CaCl_2_, 1 MgCl_2_, 25 NAHCO_3_, 1.25 NaH_2_PO_4_ and 25 D-glucose (final osmolarity 310 mmol/kg) and bubbled with carbogen (95% oxygen, 5% carbon dioxide), giving a pH of 7.4. Slices were placed in a standard ACSF-perfused bath at 32–34°C and visualized with an upright microscope (Zeiss Axioskop) using infrared-differential interference contrast optics, optimized as described previously [52]. Whole-cell current-clamp recording were made from the soma using Axoclamp 2A, 2B or Multiclamp 700B amplifiers. Glass pipettes (4–7 MΩ) were filled with intracellular solution containing (in mM): 130 K-methanesulfate, 10 HEPES, 7 KCl, 0.05 EGTA, 2 Na_2_ATP, 2 MgATP and 0.5 Na_2_GTP, titrated with KOH to pH 7.2. Compensation for the access resistance of the pipette and for the capacitance of the pipette were performed and monitored throughout the recording. Recordings were abandoned when the resistance exceeded 40 MΩ. The recorded potential and current were filtered at 3 or 10 kHz and digitized at 50 kHz. Single patch-clamp recordings were performed using the electrode for both injection of current and recording of the voltage, for the ISR and hysteresis experiments. For the dynamic IV method and fitting parameters to the model, simultaneous double patch-clamp recordings were made at the soma, using one electrode to inject the current and one to record the voltage. The current and voltage were recorded by the amplifier and acquired by using Axograph (www.axograph.com/). The traces were then imported into Igor Pro for analysis.

### Bistability and ISR analysis

To test for inverse stochastic resonance (ISR), current injection protocols were composed of series of 0.5 − 1 s periods of noise followed by 0.5 − 1 s period without noise. The injected noise waveforms were generated by an Ornstein-Uhlenbeck process:

$$\tau \frac{dx}{dt}=\mu -x+\sqrt{2\sigma^{2}\tau }N\left(0,1\right)$$ (4)

where *μ* is the mean, *σ* the variance, and *N*(0,1) a Gaussian white noise process with zero mean and variance equal to 1. For these protocols, the time constant was *τ* = 2 ms, the noise amplitude *σ* varied in the range of 0 − 500 *pA*, with a step size of Δ*σ* = 20, 50 or 100 pA. The mean changed with the holding current *I*_*in*_ = −500 − 0 pA, and was adapted for each cell to explore particularly the region of bistability. The resulting firing frequency *f* during the noise injection period and the mean noise amplitude *σ* were used to generate the ISR curve. All the curves measured with a holding current in the bistability range or higher (with non-zero firing rate at zero noise amplitude *f*(*σ* = 0) ≠ 0) were averaged and smoothed with a Gaussian filter with a width similar to the step size in noise amplitude (10 or 20 pA). The optimal noise amplitude for reduction in firing *σ*_*opt*_ was obtained for each cell as the minimum of the ISR curve, and the step noise amplitude of the stimulation provided the measurement error.

As the bistability of Purkinje cells is history dependent, it was necessary to measure the ISR curves in comparable conditions (silent or firing). We injected a noise waveform with linearly increasing and decreasing amplitudes (0.5 nA/s, S1E Fig). We analyzed the firing frequency in intervals of 200 ms, and separated intervals where the cell was firing in the previous intervals, and intervals where the cell was previously silent. For each category, we obtained the ISR curves by performing a running average (bin size = 20 pA, S1F Fig).

To measure the *f-I* curve, cells were hyperpolarized to -65 mV, a series of 30 step currents were injected (1 s, 50 pA increment), and firing frequency was calculated over the 1 s periods. To characterize the bistability of Purkinje cells, slow ramps of current were injected (0.9 nA/s), ascending for 1 s and descending for 1 s, and repeated 10 times. The cell was first hyperpolarized to -65 mV to stop firing. For each spike, the instantaneous frequency and the instantaneous injected current was calculated. The range of bistability was quantified as the difference between the frequency of the first spike (during the ascending ramp of current, *f*_*up*,_) and the last spike (during the descending ramp *f*_*down*_) Δ*f* = *f*_*up*_ − *f*_*down*_, and as the difference of injected current for the first and last spike Δ*I* = *I*_*up*_ − *I*_*down*_.

### The dynamic IV method

The dynamic IV method developed by [18] is based on a simple representation of neuronal biophysics, where subthreshold injected current (*I*_*in*_) is split into ionic transmembrane current (*I*_*m*_) and capacitive current (*I*_*C*_). In addition, the neuron receives noisy current input (*I*_*noise*_) from background synaptic activity and other sources of high frequency noise. This can be rearranged to find the ionic current flowing through the neuronal membrane. Thus, by injecting a rapidly fluctuating current *I*_*in*_ to a neuron, the relationship between ionic current and voltage, during physiological spike generation, can be found.

$$I_{m}\left(V,t\right)=I_{in}\left(t\right)-C\frac{dV}{dt}+I_{noise}$$ (5)

The injected noise current was the sum of two waveforms generated by Ornstein-Uhlenbeck processes (Eq 4), with time constants *τ*_*fast*_ = 3 ms, *τ*_*slow*_ = 10 ms (mimicking excitatory and inhibitory synaptic currents [18]). Two different amplitudes of noise were used, *σ* = 153 pA or *σ* = 235 pA, and the mean was adapted according to the injected holding current. Individual protocols were composed of 500 ms without noise followed by 20 s of the noise waveform. The membrane potential *V* was recorded in response to the noisy current injection, and the ionic transmembrane current *I*_*m*_ was calculated by subtracting the capacitive current *I*_*C*_ from the injected current *I*_*in*_ (Eq 5). The after-hyperpolarization and the initial repolarization phase of each action potential (10 *ms* after the peak) were excluded from the analysis. As the distribution of the data points is approximately Gaussian for a given voltage, the dynamic IV curve can be defined as the average of *I*_*m*_ versus *V* (Fig 3C):

$$I_{dyn}\left(V\right)=mean\left[I_{m}\left(V,t\right)\right]$$ (6)

The capacitance (used to evaluate the capacitive current) was estimated by minimizing the variance of *I*_*m*_ within individual voltage bins. Eq (5) can be transformed by inserting the estimated capacitance *C*_*e*_:

$$\frac{I_{in}}{C_{e}}-\frac{dV}{dt}=\frac{I_{m}}{C}+\left(\frac{1}{C_{e}}-\frac{1}{C}\right)I_{in}-\frac{I_{noise}}{C}$$ (7)

The variance of this function is:

$$var[{\frac{I_{in}}{C_{e}}-\frac{dV}{dt}]}_{V}=var[{\frac{I_{m}}{C}]}_{V}+{\left(\frac{1}{C_{e}}-\frac{1}{C}\right)}^{2}var{\left[I_{in}\right]}_{V}+var{\left[\frac{I_{noise}}{C}\right]}_{V}$$ (8)

When $\left(\frac{1}{C_{e}}-\frac{1}{C}\right)$ becomes zero, i.e. the estimated capacitance is correct, this variance is minimized.

The dynamic IV curve can be transformed into an integrate-and-fire (IF) type neuronal model, with voltage dynamics of the type shown in Eq (9), where *F*(*V*) is a non-linear function of voltage:

$$\frac{dV}{dt}=F\left(V\right)+\frac{I\left(t\right)}{C}$$ (9)

*F*(*V*) is related to the dynamic IV curve by Eq (10):

$$F\left(V\right)=-\frac{I_{dyn}\left(V\right)}{C}$$ (10)

The experimentally derived −*I*_*dyn*_(*V*)/*C* curve can be fitted with a function *F*(*V*) describing an exponential integrate-and-fire (EIF) model [19], containing a linear part and an exponential rise to the action potential (Fig 3D):

$$F\left(V\right)=\frac{1}{\tau_{m}}\left(E_{L}-V+\Delta_{T}\text{exp}\left(\frac{V-V^{T}}{\Delta_{T}}\right)\right)$$ (11)

The parameters are membrane time constant *τ*_*m*_, resting potential *E*_*L*_, threshold potential *V*^*T*^, and spike slope factor Δ_*T*_.

### aEIF model

The exponential integrate-and-fire model with adaptation [18] is defined by:

$$C\frac{dV}{dt}=-g_{L}\left(V-E_{L}\right)+g_{L}\Delta_{T}e^{\frac{V-V^{T}}{\Delta_{\text{T}}}}-w+I_{in}\left(t\right)$$ (12)

$$\tau_{w}\frac{dw}{dt}=a\left(V-E_{L}\right)-w$$ (13)

*If V* > *V*_*spike*_ then *V*→*V*_*r*_ and w → w + *b*

where *C* is the capacitance, *E*_*L*_ is the leak reversal potential, *g*_*L*_ is the leak conductance, *V*^*T*^ is the threshold potential, Δ_*T*_ is the spike slope factor, *V*_*r*_ is the membrane potential reset after a spike, *w* is the adaptation current, *a* is the level of subthreshold adapation, *τ*_*w*_ is the adaptation time constant and *b* is the adaptation current reset after a spike, *V*_*spike*_ is the conditional threshold for spike generation (*V*_*spike*_ = 0).

### Parameter fitting for the aEIF model

Fitting electrophysiology data of Purkinje cells to an aEIF model was achieved using a combination of the dynamic IV method and the procedure used for fitting an aEIF model to synthetic data described in [20].

#### Passive parameters

A good estimate of the membrane time constant *τ*_*m*_ of the Purkinje cell is obtained by fitting the late phase of the voltage response to a short current pulse (0.5 ms, 1 nA) [21] (S2 Fig, see S1 Text). As the aEIF remains a single compartment model, we use the approximation of the capacitance obtained using the dynamic IV method (although not optimal, as the somato-dendritic coupling of Purkinje cells is high). The reversal potential *E*_*L*_ is also best estimated using the dynamic IV method, as the cell is spontaneously active. We estimate the value for the leak conductance at the soma and proximal dendrite using the membrane time constant and the capacitance according to gL=Cτm.

#### Subthreshold adaptation

The subthreshold adaptation parameter *a* was determined as follows: when the potential *V* is fixed, the adaptation current *w* is close to *a*(*V* − *E*_*L*_). Therefore the linear part of the dynamic IV curve (apart from the exponential term) is:

$$I_{dyn}=\left(g_{L}+a\right)\left(V-E_{L}\right)$$ (14)

We obtain the parameter *a* by subtracting the leak conductance *g*_*L*_ from the slope fitted to the linear part of the dynamic IV curve.

#### Spike-triggered adaptation

To determine the contribution of spike-triggered adaptation, we use the voltage response to noise injection in a similar way as for constructing the dynamic IV curve. Far away from threshold, the adaptation current is:

$$w=-I_{in}-C\frac{dV}{dt}-g_{L}\left(V-E_{L}\right)$$ (15)

This estimate is composed of the spike-triggered adaptation *w*_*spike*_ and the subthreshold adaptation *a*(*V* − *E*_*L*_). We can express *w*_*spike*_ as:

$$w_{spike}=-I_{in}-C\frac{dV}{dt}-\left(g_{L}+a\right)\left(V-E_{L}\right)$$ (16)

We plotted the estimated *w*_*spike*_ against time since the last action potential (Fig 3E). The distribution of the data points is approximately Gaussian for a given time. Therefore we defined the time course of the spike-triggered adaptation after a spike as the average of *w*_*spike*_ versus time. The curve can be fitted by a single exponential yielding an estimate of the time constant *τ*_*w*_ and the value of the spike-triggered adaptation reset *b*, with *w*_*spike*_(*t* = 0) = *b* (Fig 3F).

### Simulations

All simulations were done in Matlab R2014b using the forward Euler method with an integration step of 0.1 ms. We confirmed that this integration step produces stable numerical results. The bifurcation and phase-plane analyses were carried out in XPPAUT 7.0. The model code is available on ModelDB (https://senselab.med.yale.edu/modeldb/).

The parameters of the aEIF model were set to the values determined by fitting data from a representative Purkinje cell. A variation of the parameters within the range occurring in the Purkinje cell population was performed to determine their influence on the ISR range. We note that all occurring parameter combinations were restricted to $\frac{a}{g_{L}}>\frac{\tau_{m}}{\tau_{w}}$, corresponding to type II excitability (Fig 4D). The representative parameters were:

*C* = 268 pF, *E*_*L*_ = −51.31 mV, *V*^*T*^ = −53.23 mV, Δ_*T*_ = 0.85 mV, *g*_*L*_ = 8.47 nS, *a* = 37.79 nS, *b* = 441.12 pA, *τ*_*w*_ = 20.76 ms.

Noise stimulus was modeled as I(*t*) = *I*_*mean*_ + *I*_*noise*_(*t*), where *I*_*mean*_ is constant and *I*_*noise*_(*t*) is current noise with zero mean generated using an Ornstein-Uhlenbeck process with amplitude *σ* and time constant τ_c_ = 2 ms.

Noise stimulus with an additional timed excitatory synaptic input was modeled as *I*(*t*) = *I*_*mean*_ + *I*_*noise*_(*t*) + *I*_*syn*_(*t*), where *I*_*syn*_(*t*)is the biexponential excitatory synaptic input, described as the solution of the equation [53]:

$$\tau_{1}\tau_{2}\frac{d^{2}}{dt^{2}}I_{syn}+\left(\tau_{1}+\tau_{2}\right)\frac{d}{dt}I_{syn}+I_{syn}=Am\left(1-I_{in}\right)\delta \left(t-t_{st}\right)/K\left(\tau_{1,}\tau_{2}\right)$$

where *t*_*st*_ is the stimulation time, *Am* is the amplitude of the stimulus, and

$$K\left(\tau_{1,}\tau_{2}\right)=\frac{1}{\tau_{2}-\tau_{1}}\left[{\left(\frac{\tau_{2}}{\tau_{1}}\right)}^{\frac{\tau_{1}}{\tau_{1}-\tau_{2}}}-{\left(\frac{\tau_{2}}{\tau_{1}}\right)}^{\frac{\tau_{2}}{\tau_{1}-\tau_{2}}}\right]$$ (17)

with rise time constant *τ*_1_ = 1.5 ms and decay time constant *τ*_2_ = 10 ms.

### ISR range

To estimate the ISR range in the parameter space, we rescaled the aEIF model in the following way [16]:

$$\frac{d\bar{V}}{d\bar{t}}=-\bar{V}+e^{\bar{V}}-\bar{w}+\bar{I}$$ (18)

$$T\frac{d\bar{w}}{d\bar{t}}=-A\bar{V}-\bar{w}$$ (19)

where *T* = *τ*_*w*_/*τ*_*m*_, *A* = *a*/*g*_*L*_, $\bar{I}=\frac{I}{g_{L}\Delta_{T}}+\left(1+\frac{a}{g_{L}}\right)\frac{\left(E_{L}-V^{T}\right)}{\Delta_{T}}$, $\bar{t}=\frac{t}{\tau_{m}}$, $\bar{b}=\frac{b}{g_{L}\Delta_{T}}$, $\bar{V_{rest}}=\frac{V_{reset}-V^{T}}{\Delta_{T}}$, $\bar{V}=\frac{V-V^{T}}{\Delta_{T}}$, $\bar{w}=\frac{w+a\left(E_{L}-V^{T}\right)}{g_{L}\Delta_{T}}$.

The bistability range in the model is defined similarly as for the recorded Purkinje cells, by the following expression: Δ*I = I*_*up*_ − *I*_*down*_, where *I*_*up*_ corresponds to the minimal current needed to elicit a spike when the model starts from the rest state and *I*_*down*_ is the maximal current at which the spiking stops given that the model starts in the periodic spiking state (Fig 4D). The value of *I*_*up*_ is referred to as the rheobase current, and for the aEIF model possessing type II excitability it has the following analytical expression [16]:

$$I_{up}=\left(g_{L}+a\right)\left[V^{T}-E_{L}-\Delta_{T}+\Delta_{T}log\left(1+\frac{\tau_{m}}{\tau_{w}}\right)\right]+\Delta_{T}g_{L}\left(\frac{a}{g_{L}}-\frac{\tau_{m}}{\tau_{w}}\right)$$ (20)

#### Probability of spiking

The probability of spiking is proportional to the fraction of time the trajectory spends in the region of V-w phase space leading to a spike. We measured the amount of time *t*_*rest*_ the aEIF model has spent in the basin of attraction of a stable fixed point. Then the probability of being in the rest state is calculated as $P_{r}=\frac{t_{rest}}{t}$, where *t* is the total integration time. There are only two states in the phase space of aEIF model: spiking and rest. Therefore the probability of spiking is calculated as *P*_*sp*_ = 1 − *P*_*r*_.

For simulations with Ornstein-Uhlenbeck noise, the probabilities and averaged firing rates were calculated after 20 repetitions with duration of 30 *s* each. We tested longer simulations and found that 30 *s* ensures an accurate and stable estimate. For simulations with excitatory biexponential input (Eq 17), the probabilities *P*_*sp*_ were calculated in bins of 20 ms in 1000 sweeps with a duration of 6 s each.

#### Mutual information rate

To calculate the mutual information rate (MI) between the input and output spike trains we used the context tree weighting algorithm as described in [54]. We simulated the aEIF model, extracted the input and output spike times, and then used the algorithm with bin size *b* = 25 ms and depth parameter *D* = 40 bins, corresponding to a time window of 1 s. The total duration of a sweep was 1,000 s. The algorithm was used 10 times for each data point to provide a reliable estimate of mutual information. We confirmed that the MI estimates converge by increasing the depth parameter D and the sweep duration.

## Supporting Information

S1 Text The effect of dendrites on ISR in Purkinje cells. (DOCX)

S1 Fig History dependence of the ISR curve. A. Current injection of 1 *s* noise waveform periods in a Purkinje cell in a cerebellar slice, as in Fig 1A. B. Firing frequency vs. noise amplitude *σ* for five different holding currents I_in_. C. Current injection of 1 *s* noise waveform periods in a different cell with a more pronounced bistability. The firing frequency during each noise period is influenced by the initial state of the cell (firing or silent). D. Firing frequency vs. noise amplitude *σ* for three different holding currents I_in_. E. Current injection of noise waveform with linearly increasing and decreasing amplitude. Periods of 200 *ms* duration were separated according to the state of the cell (firing or silent) in the previous interval. F. Firing frequency vs. noise amplitude *σ* for the two categories. Continuous curves are running averages.
(EPS)

S2 Fig ISR and dendrite filtering. A. Experimental determination of dendritic filtering properties. Voltage response of a Purkinje cell (black) to a short current pulse (0.5 ms, 1 nA), fitted with a biexponential function with time constants *τ*_*m*_ and *τ*_*c*_ (red). B. Mean firing rate in the experiment and the aEIF model in response to current noise stimulation, using the estimated dendrite filter parameters, *g*_*c*_ = 10.2 nS. C. Mean firing rate of the aEIF model with optimized *g*_*c*_ = 7.5 nS to quantitatively match the experimental ISR.
(EPS)

S3 Fig ISR in a detailed Purkinje cell model. A. Top, somatic voltage recording from a detailed Purkinje cell model [22] during injection of the noisy current waveform shown at the bottom (similar to the stimulus used in Fig 1A, but with a different range of noise amplitudes). B. Averaged firing frequency (5 simulations) during 1 *s* noise waveform periods vs noise amplitude *σ* at zero holding current. The model shows ISR with optimal noise amplitude between 120 and 150 pA.
(EPS)

S4 Fig Mutual information and spiking response for high intensity signal input. A. Mutual Information rate of the input and output spike train in the aEIF model when stimulated with 5 Hz signal input. B. Continuous voltage response of the aEIF model when stimulated by 30 pA noise and a Poisson spike train (input amplitude 100 pA, mean frequency 5 Hz, duration 180 seconds). C. Recording of the membrane potential of a Purkinje cell in the awake cat (duration, 180 seconds; adapted from [9]).
(EPS)

S5 Fig Membrane potential distribution during spiking and silent states. A. Membrane potential distributions computed from a somatic whole-cell patch-clamp recording from a Purkinje cell during a stimulus, which evokes transitions between spiking and silent states (Fig 1A). B. Membrane potential distributions in the aEIF model. C. Somatic membrane potential distributions in the De Schutter and Bower model (see [22]).
(EPS)
