# Supplementary material for: Inverse Stochastic Resonance in Cerebellar Purkinje Cells
Source: PLoS Comput Biol. 2016 Aug 19;12(8):e1005000. doi: 10.1371/journal.pcbi.1005000 (PMC4991839; doi:10.1371/journal.pcbi.1005000)
Supplement: S1 Text — (DOCX) [file pcbi.1005000.s001.docx]

**The effect of dendrites on ISR in Purkinje cells**

For all simulations in the main text we use the single-compartment aEIF model. Using the dynamic IV method (see Materials and Methods), we estimated the parameters of the aEIF model for different Purkinje cells. However, fitting of the parameters of an aEIF model based on data obtained with somatic recordings describes mostly the somatic properties of a neuron. This leads to the discrepancy between the optimal noise levels for the single-compartment model and actual data (compare Fig 1B and Fig 4B; model based on recordings from the same cell). This discrepancy is expected because the single-compartment aEIF model does not include the dendrite compartment. Since they have an important role in in the biophysics of Purkinje cells, here we determine the implications of including the dendrites in a reduced model and evaluate the role of dendrites for the ISR phenomenon.

While it is a single-compartment model, we want to take advantage of the aEIF model to allow phase-plane analysis, rather than fitting a two-compartment model or using the biophysically detailed approach, which could not be studied analytically. To compensate for the absence of a dendrite in the aEIF model, we add a passive dendrite compartment to it. Then we estimate the parameters of the dendrite using the impulse response of a Purkinje cell, use these parameters in the aEIF model, and show that ISR is still present.

*Two-compartment model*

To evaluate the role of dendrites in the aEIF model, we first consider a two-compartment model in the passive regime. It makes it analytically easier to start with this passive case that corresponds to the subthreshould behavior of the membrane potential. Then we will use this estimation in the following subsection.

Here are the two equations describing the current balance in the somatic and dendritic compartment of the model:

(S1)

where corresponds to the somatic voltage and is the voltage of the dendritic compartment, and where is the membrane capacitance. The parameter represents the somatic surface area as a fraction of the total surface area of the soma and dendrites, +, is the coupling conductance between the compartments and is the leak conductance. Since (S1) is a system of linear differential equations, it can be solved analytically by subtraction of the second equation from the first, followed by integrating the system:

(S2)

where and . This describes the voltage difference between the somatic and dendritic compartments in the subthreshold regime.

The following dendrite estimation is strictly valid only in the subthreshold regime. When the voltage goes beyond the threshold it becomes incorrect. But given that most of the time the membrane potential is subthreshold (Fig 5), and the action potential upstroke is very fast because of the exponential term, we assume here that this approximation is sufficient to capture the main effect of a passive dendrite.

*Estimation of dendrite parameters*

To estimate the parameters of the Purkinje cell dendrite, we approximate the cell as a two-compartment model as described in the previous section. Then we aim to determine the kernel response to an infinitely short current injection as in [1]. Experimentally, we inject a brief current pulse (duration dur = 0.5 ms and amplitude 1 nA) into the soma and measure the somatic voltage response, which is then fitted by the kernel response (S2A Fig). This kernel can be derived analytically from (S1) in the following form (for details see [2]):

(S3)

where is the membrane time constant, is the coupling time constant, is the somatic capacitance, is the duration of the pulse, and p is the relative somatic surface area.

We fit the experimentally measured voltage response with this biexponential kernel with (C_m_, p, , ) as free parameters. Since the Purkinje cell has a large dendritic tree, the single pulse response consists of two phases: fast equilibration of membrane potential between the somatic and dendritic compartment via the coupling conductance, and slow discharging of the membrane potential in the entire neuron towards the resting potential via the leak conductance. The coupling conductance between two compartments is then defined as.

Table of parameters:

| $C_{m}$ | $\tau_{S}$ | $\tau_{C}$ | $p$ | *dur* | *I* |
| --- | --- | --- | --- | --- | --- |
| 66.97 pF | 32.91 ms | 0.70 ms | 0.07 | 0.5 ms | 1 nA |

*The effect of dendrites on ISR*

As mentioned above, the aEIF model contains only one single compartment. In this section we use the passive dendrite model derived in the previous section and include it into the aEIF model. To achieve this, we add the dendritic term that represents the passive filtering property of the dendritic compartment, as described in (S1-2):

(S4)

(S5)

As it is not possible to experimentally determine all the parameters of the spiking aEIF model and the dendritic parameters of a Purkinje cell at the same time, we follow an *ad hoc* strategy: we use both the parameters of the simple aEIF model fitted to data from a Purkinje cell (see Materials and Methods) and the dendritic parameters determined for the same cell, as described above.

The addition of an effective dendrite compartment brings an additional current to the aEIF model as is replaced by

(S6)

The additional current represented by the integral term significantly changes the total input to the cell. It provides a current sink, which is dependent on the input. This significantly reduces the total amount of input current. It becomes necessary to compensate for this current sink to keep the model within the same ISR range as the aEIF model without a dendrite.

*Dendrite compensation*

We are motivated by the fact that in the experiment it is relatively easy to change the mean of the input current compared to changing other parameters. Therefore we develop a compensation model that keeps the aEIF model tuning in the same bistability range as if the dendrite was not present.

First let us consider the external input to the aEIF model (eq. S4) that can be decomposed into two components:

(S7)

where corresponds to the constant current while is the noise part, with Ornstein-Uhlenbeck noise . We use the following analytical expression to keep the model within the same mean of an input current tuned for ISR:

(S8)

where is the total conductance. Thus, the compensated input to the aEIF model will be

(S9)

After this compensation, the aEIF model with a dendrite is again able to display ISR because the bistability range of input current is preserved (S2B Fig). Addition of a dendrite with compensation indeed changes the shape of the ISR curve. These changes are expected from the compensation model we use. The mean of a compensated input (S6) is tuned in the bistability regime. But the variance of the noise component stays the same. This leads to changes in variance tuning because the total input to the model including noise will be filtered by the dendrite. It results in a smaller effective noise variance because of the attenuation by the dendrite, and shifts the ISR region to the right since the effective noise amplitude will be lower (S2B Fig ISR).

Unfortunately, fitting an aEIF model using the dynamic IV procedure is not perfect because it takes into account not only the somatic properties of a cell, but partially also the dendrite [18]. To separately estimate the dendrite parameters of the Purkinje cell we use the impulse response of the cell (S2A Fig). Then we use the aEIF model with mean current compensation and dendrite parameters estimated from the experiment. As a consequence, the dendrite effect is taken into account twice – using the dynamic IV fitting and the impulse response of the cell. This leads to the quantitative mismatch between the experimentally measured ISR and model ISR (S2B Fig). Since the dendrite is overestimated in the model, we reduce the coupling conductance from 10.2 nS to 7.5 nS (S2C Fig). This allows a more precise quantitative match between the experimental and model ISR.

In the previous sections we have shown that ISR is still present in the aEIF model even if the effects of a dendrite are included. But the mean input current has to be rescaled according to the dendrite parameters. Indeed, addition of the dendrite changes the tuning for the ISR, shifting the optimal noise amplitude to values more similar to those observed experimentally. It is likely that fitting the parameters of a two-compartment or multicompartmental model to the data would probably lead to an even better quantitative match between the experiment and the model.

**Supporting Information References**

1. Clopath C, Jolivet R, Rauch A, Lüscher HR, Gerstner W. Predicting neuronal activity with simple models of the threshold type: adaptive exponential integrate-and-fire model with two compartments. Neurocomputing. 2007 Jun;70(10-12):1668–73.

2. Gerstner W, Kistler W. Spiking neuron models: single neurons, populations, plasticity. Camb Univ Press. 2002.

**S1 Fig. History dependence of the ISR curve.**

A. Current injection of $1 s$ noise waveform periods in a Purkinje cell in a cerebellar slice, as in Fig 1A. B. Firing frequency vs. noise amplitude $\text{σ}$ for five different holding currents I_in_. C. Current injection of $1 s$ noise waveform periods in a different cell with a more pronounced bistability. The firing frequency during each noise period is influenced by the initial state of the cell (firing or silent). D. Firing frequency vs. noise amplitude $\text{σ}$ for three different holding currents I_in_. E. Current injection of noise waveform with linearly increasing and decreasing amplitude. Periods of 200 *ms* duration were separated according to the state of the cell (firing or silent) in the previous interval. F. Firing frequency vs. noise amplitude $\text{σ}$ for the two categories. Continuous curves are running averages.

**S2 Fig. ISR and dendrite filtering.**

A. Experimental determination of dendritic filtering properties. Voltage response of a Purkinje cell (black) to a short current pulse ($0.5 \mathrm{ms}$, $1 \mathrm{nA}$), fitted with a biexponential function with time constants $\tau_{m}$ and $\tau_{c}$ (red). B. Mean firing rate in the experiment and the aEIF model in response to current noise stimulation, using the estimated dendrite filter parameters, $g_{C}=10.2 \mathrm{nS}$. C. Mean firing rate of the aEIF model with optimized $g_{C}=7.5 \mathrm{nS}$ to quantitatively match the experimental ISR.

**S3 Fig. ISR in a detailed Purkinje cell model.**

A. Top, somatic voltage recording from a detailed Purkinje cell model [22] during injection of the noisy current waveform shown at the bottom (similar to the stimulus used in Fig 1A, but with a different range of noise amplitudes). B. Averaged firing frequency (5 simulations) during 1 *s* noise waveform periods vs noise amplitude $\text{σ}$ at zero holding current. The model shows ISR with optimal noise amplitude between 120 and 150 pA.

**S4 Fig. Mutual information and spiking response for high intensity signal input.**

A. Mutual Information rate of the input and output spike train in the aEIF model when stimulated with 5 Hz signal input. B. Continuous voltage response of the aEIF model when stimulated by 30 pA noise and a Poisson spike train (input amplitude 100 pA, mean frequency 5 Hz, duration 180 seconds). C. Recording of the membrane potential of a Purkinje cell in the awake cat (duration, 180 seconds; adapted from [9]).

**S5 Fig. Membrane potential distribution during spiking and silent states.**

A. Membrane potential distributions computed from a somatic whole-cell patch-clamp recording from a Purkinje cell during a stimulus, which evokes transitions between spiking and silent states (Fig 1A). B. Membrane potential distributions in the aEIF model. C. Somatic membrane potential distributions in the De Schutter and Bower model (see [22]).
